# Fortification of FeS Clusters Reshapes Anaerobic CO Dehydrogenase into an Air‐Viable Enzyme Through Multilayered Sealing of O_2_ Tunnels

**DOI:** 10.1002/anie.202508565

**Published:** 2025-06-12

**Authors:** Suk Min Kim, So Yeon Kong, Jingu Kang, Jeong Seok Ji, Sung Heuck Kang, Hye‐Jin Yoon, Hyunwoo Kim, Jungki Ryu, Hyung Ho Lee, Yong Hwan Kim

**Affiliations:** ^1^ Department of Biotechnology The Catholic University of Korea Bucheon 14662 Republic of Korea; ^2^ Department of Chemistry College of Natural Sciences Seoul National University Seoul 08826 Republic of Korea; ^3^ School of Energy and Chemical Engineering Ulsan National Institute of Science and Technology (UNIST) Ulsan 44919 Republic of Korea; ^4^ Graduate School of Carbon Neutrality Ulsan National Institute of Science and Technology (UNIST) Ulsan 44919 Republic of Korea

**Keywords:** CO dehydrogenase, Enzyme engineering, FeS clusters, O_2_ sensitivity, Tunnel engineering

## Abstract

The inherent O_2_ sensitivity of Ni─Fe carbon monoxide dehydrogenases (CODHs), crucial for rapid CO to CO_2_ interconversion, presents substantial challenges for industrial application. Transforming CO/CO_2_, a prevalent anthropogenic air pollutant, into valuable carbon chemicals either directly or through intermediate steps via biocatalytic methods offers a promising pathway to achieve net‐zero emissions across industries and the environment. However, completely eliminating oxygen from industrial biotransformations, especially under ambient conditions, is exceedingly onerous. Here, we engineered variants of the CODH2 from *Carboxydothermus hydrogenoformans* (*Ch*CODH2) with dual blocking at both the O_2_ entrance and near the active site, effectively sealing the tunnel against atmospheric O_2_ levels (20%). The O_2_‐tunnel engineered A559W/V610H variant demonstrated a marked improvement in air stability, with a half‐life of 24.6 h compared to the wild type's 2.4 h. Crystallographic snapshots of this air‐viable variant after 24 h of exposure revealed the robust integrity of the fortified FeS and NiFeS clusters. Additionally, electro‐enzymatic reactions corroborated its CO/CO_2_ conversion capability even in ubiquitous air. These findings, which address the O_2_ sensitivity of anaerobic enzymes caused by O_2_‐induced metal cluster collapse, enhance their potential for biological CO/CO_2_ transformations in O_2_‐rich environments, thereby broadening their industrial viability and applicability.

## Graphical abstract

Fortification of FeS clusters in Ni─Fe CO dehydrogenase (*Carboxydothermus hydrogenoformans*) reshapes this anaerobic enzyme into an air‐viable variant through multilayered O_2_ tunnel sealing. The engineered enzyme retains structural integrity and catalytic efficiency in ambient air, offering a practical solution for industrial CO/CO_2_ bioconversion processes without the need for strict anaerobic conditions.

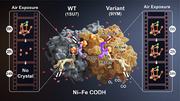

## Introduction

Molecular oxygen (O_2_), essential for energy production in obligate aerobes,^[^ ^1^ ^]^ also acts as a disruptive effector of several metal‐containing enzymes involved in the Wood–Ljungdahl pathway,^[^ ^2^, ^3^ ^]^ including carbon monoxide dehydrogenase (CODH), formate dehydrogenase, and hydrogenase. Their oxygen vulnerability arises primarily from the oxidation of metallic cofactors (e.g., Ni and Fe) at their active sites,^[^ ^2^, ^4^, ^5^ ^]^ or from the generation of reactive oxygen species and radicals due to high oxygen reactivity, leading to enzyme inactivation or damage.^[^ ^6^ ^]^ These enzymes are adapted to anaerobic, or microaerophilic conditions, and their sensitivity poses an arduous challenge for industrial applications. Enhancing oxygen tolerance is crucial for the economic and efficient expansion of enzyme‐based reactions across various industrial environments. CODH, which catalyzes reversible interconversion of toxic but valuable carbon monoxide to carbon dioxide (CO_2_), plays a vital role in environmentally friendly technologies and carbon resource utilization.^[^ ^7^, ^8^, ^9^ ^]^ Operating these enzymes in ambient air conditions eliminates the need for complex anaerobic setups and reduces additional costs, thereby making enzyme‐catalyzed technologies^[^ ^10^, ^11^, ^12^ ^]^ more accessible and cost‐effective.

Unlike the slow Mo─Cu CODHs that exhibit low O_2_ sensitivity in aerobic CO conversion,^[^ ^13^, ^14^ ^]^ Ni─Fe CODHs in anaerobic CO conversion represent a class of diffusion‐limited extremely active enzymes^[^ ^15^, ^16^ ^]^ (Table S1), which are valuable due to their robust expression and straightforward production in standard expression systems.^[^ ^17^ ^]^ Moreover, Ni─Fe CODHs demonstrate resistance to various inhibitory gases at industrially relevant concentrations, making them viable for use with actual industrial off‐gases that contain gas impurities (such as CN^−^, H_2_S, and SO_x_).^[^ ^18^, ^19^ ^]^ Indeed, the feasibility of pilot‐scale implementation of actual CO gas conversion through integrated reactions with formate dehydrogenase emphasizes the industrial potential of biocatalysts.^[^ ^18^, ^19^ ^]^ However, expanding their use in O_2_‐rich industrial environments^[^ ^20^ ^]^ and applications such as electrochemical CO_2_ reducing reactions^[^ ^21^ ^]^ necessitates biocatalysts that can exhibit oxygen tolerance up to ambient air levels—a common and critical challenge for most metal‐containing gas‐utilizing enzymes, including CODH.

In this article, we engineered an air‐viable CODH beyond O_2_‐tolerant CODH variants by systematically sealing the O_2_ tunnels adjacent to the active site, thus obstructing oxygen ingress, preserving metal FeS clusters, and resulting in dramatically increased oxygen tolerance to unnatural levels. These modifications are designed to overcome the destructive effects of oxygen while enhancing the enzyme's catalytic capacities in O_2_‐rich environments. X‐ray structural analysis of the engineered variants provides insights into the mechanisms of enhanced O_2_ tolerance through retarding the collapse of O_2_‐vulnerable FeS clusters. Furthermore, electrochemical assays have been conducted to explore the applicability of these tunnel‐modified variants for CO gas conversion in ambient conditions, demonstrating their potential for broad industrial applications.

## Results and Discussion

### Dual Blocking of Tunnels Responsive to Putative O_2_ Transport in *Ch*CODHs

**Figure 1:**
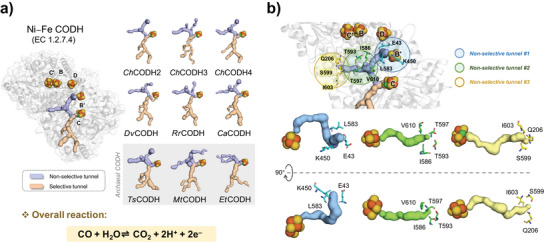
Gas transfer tunnels in Ni─Fe CODHs. a) Gas tunnels for gaseous molecule transfer in Ni─Fe CODHs. Two types are indicated: non‐selective tunnels (putative O_2_/H_2_O) and selective tunnels (putative CO/CO_2_). The nine representative protein structures are *Ch*CODH2 (*Ch*, *Carboxydothermus hydrogenoformans*; PDB 1SU7),^[^ ^23^ ^]^ *Ch*CODH3 (PDB 7ZKJ),^[^ ^24^ ^]^ *Ch*CODH4 (PDB 6ELQ),^[^ ^15^ ^]^ *Ca*CODH (*Ca*, *Clostridium autoethanogenum*; PDB 6YU9),^[^ ^25^ ^]^ *Dv*CODH (*Dv*, *Desulfovibrio vulgaris*; PDB 6OND),^[^ ^26^ ^]^ *Et*CODH (*Et*, *Candidatus Ethanoperedens thermophilum*; PDB 8RIU),^[^ ^27^ ^]^ *Mt*CODH (*Mt*, *Methanosarcina themophila*; PDB 9C0R),^[^ ^28^ ^]^ *Rr*CODH (*Rr*, *Rhodospirillum rubrum*; PDB 1JQK),^[^ ^29^ ^]^ and *Ts*CODH (*Ts*, *Thermococcus* sp. AM4; PDB 6T7J).^[^ ^30^ ^]^ *Ch*CODH3, *Ca*CODH, and *Mt*CODH, with slightly modified non‐selective tunnels, are subunits of the CODH/ACS complex, indicating potential adaptations for their roles. Archaeal models are displayed in a gray box. b) Non‐selective tunnels in *Ch*CODH2. The three entrances (#1–#3), related to O_2_ transfer, are displayed. Tunnel‐forming residues are shown with colored circles: blue (tunnel #1), green (tunnel #2), and orange (tunnel #3), arranged to reflect their spatial influence on O_2_ migration.

Ni─Fe CODHs, which exhibit highly similar 3D structures to each other, commonly feature tunnels through which molecules such as CO and CO_2_ can be transported. These tunnels are broadly categorized into selective and non‐selective types. Recently, we proposed that oxygen might migrate through one of these, the “non‐selective tunnel”,^[^ ^8^, ^22^ ^]^ where water molecules are also found (Figure 1a). To enhance the oxygen tolerance of CODH enzymes, it is essential to restrict O_2_ movement within these tunnels to protect the active center (Ni─FeS cluster). Thus, we proposed a strategy to block these tunnels. As shown in Figure 1b, the non‐selective tunnel comprises three main pathways. We hypothesized that blocking the entrances of these tunnels (non‐selective tunnel #1–3), where oxygen molecules enter, could further improve the enzyme's oxygen resistance (Figure S1). It was observed that the non‐selective tunnel acts as a pathway for O₂ migration.^[^ ^8^ ^]^ Our approach focused on modifying key residues at the tunnel entrances (non‐selective tunnel #1–3) as follows: E43, K450, and L583 for non‐selective tunnel #1; I586, T593, T597, and V610 for non‐selective tunnel #2; and Q206, S599, and I603 for non‐selective tunnel #3. These residues were substituted with bulky and charged histidine residues to obstruct oxygen passage. Consequently, various mutants were generated through heterologous expression in *Escherichia coli* BL21(DE3) strains (Table S2). Each mutant exhibited distinct characteristics at the bottleneck region and showed reduced O_2_ sensitivity, providing insights into the role of the three tunnels (Table S3).

**Figure 2:**
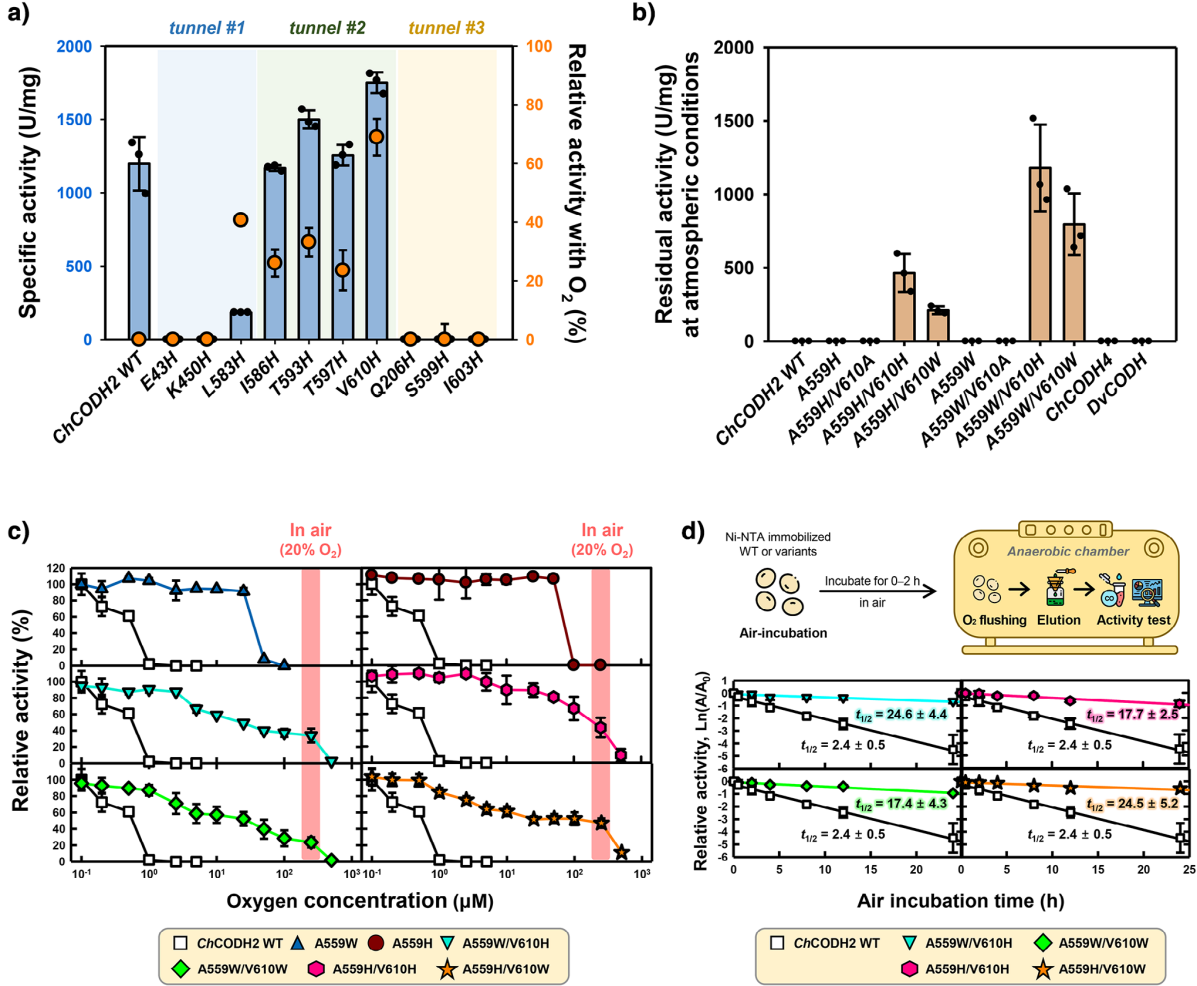
Air viability of *Ch*CODH2 and variants. a) Specific activities of histidine‐substituted *Ch*CODH2 variants and their relative activities with O_2_. CO oxidation catalyzed by *Ch*CODH2 variants in the absence (blue bar) or presence (orange circle) of O_2_. For O_2_ conditions, enzymes were preincubated in HEPES buffer containing 10 µM O_2_ for 2 min before assay. Data are mean ± S.D. (*n* = 3). 1 U/mg = 1 µmol reduced viologen·min^−1^·mg^−1^ b) Residual CO oxidation activity of less O_2_‐sensitive Ni─Fe CODHs and engineered *Ch*CODH2 under atmospheric O_2_. Data are mean ± S.D. (*n* = 3). c) O_2_ sensitivity profiles of air‐viable *Ch*CODH2 variants at different O_2_ concentrations: WT (squares), A559H (circles), A559W (triangles), A559H/V610H (hexagons), A559H/V610W (stars), A559W/V610H (reverse triangles), A559W/V610W (diamonds). Salmon bars indicate atmospheric O_2_ (−250 µM). Data are mean ± S.D. (*n* = 3). See Supplemental Experimental Procedures. d) Time course of residual activity under air. *Ch*CODH2 (squares) and variants were exposed to air up to 24 h and re‐purified under anaerobic conditions via Ni‐NTA resin. Data are mean ± S.D. (*n* = 3).

Figure 2a shows the activity changes of histidine‐substituted mutant enzymes in the presence and absence of oxygen (10 µM O_2_). The specific activity of the mutants in tunnel #2, compared to the *Ch*CODH2 (*Ch*, *Carboxydothermus hydrogenoformans*) wild type (WT; 1200 U⋅mg^−1^), did not show any significant decrease: 1170 U⋅mg^−1^ for I586H, 1500 U⋅mg^−1^ for T593H, 1260 U⋅mg^−1^ for T597H, and 1750 U⋅mg^−1^ for V610H. In contrast, the mutants in tunnels #1 and #3, except for L583H (180 U⋅mg^−1^), exhibited almost no activity, indicating the critical role of these positions in CODH enzyme function. Energy predictions showed that inactive variants (E43H, K450H, Q206H, S599H, and I603H) had less favorable stability scores than active ones (T597H, I586H, and T593H) (Table S4), suggesting impaired folding or structural integrity. Upon exposure to dissolved O_2_ (10 µM), the residual activities of the variants in tunnel #2 were highest for V610H (70%), followed by T593H, I586H, and T597H (24%–33%), while WT showed no residual activity (0%). Sequence alignment (Figure S2) shows that the substituted residues are not highly conserved, implying limited impact on activity. These results reveal that the tunnel entrance residues affect oxygen sensitivity and that the tunnel‐forming residues in tunnel #2 are highly involved in oxygen transport, suggesting a close relationship between oxygen sensitivity and these residues.

Building on these findings, we employed the most promising variant, V610H, alongside the previously reported less O_2_‐sensitive A559 mutants to evaluate their combined effect under atmospheric conditions (250 µM with approximately 20% oxygen) (Figure 2b). The single mutants A559H and A559W were originally tested under lower oxygen concentrations and did not exhibit any residual activity at 20% oxygen due to their sensitivity. In contrast, double mutants demonstrated considerable residual activity even under these challenging atmospheric conditions, showing a synergistic effect: the residual activities of A559H/V610H, A559H/V610W, A559W/V610H, and A559W/V610W were 460, 210, 1180, and 800 U·mg^−1^, respectively. However, introducing the small residue alanine at position 610 (A559H/V610A and A559W/V610A) resulted in negligible residual activity. Therefore, these observations suggest that introducing bulky charged or hydrophilic residues at critical positions instead of small ones can help repel O_2_. Combined with internal bottleneck tunnel modifications, this strategy improves oxygen sensitivity.

### Less O_2_ Sensitivity and Kinetics of *Ch*CODH Variants

Expanding upon the initial screening by specific oxygen levels, we conducted a comprehensive analysis across a broad range of oxygen concentrations (0–250 µM) (Figure 2c). While previously reported less O_2_‐sensitive mutants A559W and A559H showed a rapid decrease in activity above 25 µM oxygen, these engineered variants maintained residual activity under atmospheric conditions, although with a gradual decrease as oxygen concentration increased (Figure S3). Moreover, the double mutants' inhibitory oxygen concentration (IC) values markedly increased compared to the WT and single mutants A559H and A559W, indicating improved activity as oxygen concentration increased (Table S3). Notably, the near‐maximum inhibitory oxygen concentration (IC_90_) values showed all double mutants, except A559H/V610W, exceeded atmospheric levels (∼250 µM), with 450–850‐fold higher O_2_ tolerance than WT (IC_90_ 1 µM). The strategic double placements at positions A559 and V610 in *Ch*CODH2 enhanced oxygen tolerance without detrimental impacts on enzyme performance.

To explore the impact of additionally implemented tunnel bottlenecks on substrate affinity, we measured the catalytic properties of the enzymes using CO and ethyl viologen (EV) as substrates (Tables 1 and S5). The apparent Michaelis constant (*K*_m_) for CO in *Ch*CODH2 WT was previously reported to be 20 µM.^[^ ^8^ ^]^ For the variants, the *K*_m_ values varied, indicating different affinities for CO. Notably, A559H/V610H exhibited the highest *K*_m_ value of 91 µM, suggesting a decreased affinity for CO compared to *Ch*CODH2 WT. In terms of specific activity, A559W/V610H demonstrated the highest specific activity for both CO (2800 U·mg^−1^) and EV (2800 U·mg^−1^), reflecting improved catalytic efficiency. In contrast, *Ch*CODH2 WT showed specific activities of 1300 U·mg^−1^ for CO and 900 U·mg^−1^ for EV. The apparent normalized turnover number (*k*_cat_^n^) for CO was highest in A559W/V610H at 9200 s^−1^, which is 2.6 times higher than the 3600 s^−1^ observed for *Ch*CODH2 WT. These results suggest that the CO transport was not impeded by the newly introduced bottleneck at positions 559 and 610, which implies that these non‐selective tunnels might not be the main pathways for CO transport. This supports previous studies suggesting the existence of a distinctive CO‐specific tunnel.^[^ ^8^, ^31^ ^]^ The normalized catalytic efficiency (*k*_cat_^n^/*K*_m_) for CO was 100000 s^−1^·mM^−1^ in A559W/ V610H, comparable to 126000 s^−1^·mM^−1^ in A559W. Similarly, A559H/V610H showed a catalytic efficiency of 55000 s^−1^·mM^−1^ for CO, close to the 69000 s^−1^·mM^−1^ observed for A559H. These results indicate that, despite restricting oxygen movement, there was no significant impact on the substrate properties of the enzyme, suggesting that the mechanism of CO oxidation by CODH is unaffected by the mutations.

**Table 1:** Catalytic properties of *Ch*CODH variants.

CODH variant	Substrate	Ni content^a)^ (Ni mol/enzyme mol)	*K*_m_ (mM)	*k*_cat_^n^ ^b)^ (s^−1^)	*k*_cat_^n^/*K*_m_ (s^−1^·mM^−1^)
*Ch*CODH2	CO	0.42 ± 0.07	0.020 ± 0.002	3600 ± 160	179000 ± 19600
	EV		1.8 ± 0.1	2400 ± 38	1300 ± 77
A559H	CO	0.55 ± 0.05	0.061 ± 0.002	4200 ± 69	69000 ± 2500
	EV		2.1 ± 0.1	4200 ± 130	2000 ± 110
A559W	CO	0.42 ± 0.02	0.034 ± 0.002	4300 ± 120	126000 ± 8200
	EV		2.0 ± 0.1	5000 ± 93	2500 ± 130
A559H/V610H	CO	0.70 ± 0.07	0.082 ± 0.008	4400 ± 220	55000 ± 5900
	EV		1.7 ± 0.4	3200 ± 290	1900 ± 470
A559H/V610W	CO	0.50 ± 0.03	0.052 ± 0.012	3000 ± 290	57000 ± 14000
	EV		2.1 ± 0.3	3000 ± 170	1400 ± 220
A559W/V610H	CO	0.51 ± 0.01	0.091 ± 0.021	9200 ± 1000	100000 ± 26000
	EV		2.7 ± 0.5	7300 ± 590	2700 ± 540
A559W/V610W	CO	0.53 ± 0.01	0.058 ± 0.017	5000 ± 610	85000 ± 27000
	EV		1.8 ± 0.2	5500 ± 210	3100 ± 360

Kinetic data were assayed at 30 °C, pH 8. Parameters were estimated by nonlinear hyperbolic regression (SigmaPlot 10) of initial rates at seven EV concentrations (0.5–8 mM) and five CO concentrations (0.01–0.16 mM). All enzymatic specific activities (1 U/mg = 1 µmol reduced viologen·min^−1^·mg^−1^) were determined in triplicate (see Methods). Most values are rounded to the nearest 100. The relative activities of WT, A559H, and A559W are from the same published data, reproduced with permission (reference,^[^ ^8^ ^]^ Copyright 2022, *Springer Nature*).

^a)^ Ni contents refer to the molar ratio of nickel to enzyme based on total protein concentration. Values are the means ± standard deviation, *n* = 3.

^b)^ The normalized turnover number has been calculated by dividing the turnover by the Ni content.

To determine whether the activities of the variants are maintained in actual air, we observed the changes in residual activity over 24 h using Ni─NTA agarose immobilized enzymes (Figures 2d, S4, and S5). Compared to the initial activity at 0 h, the half‐life times (*t*_1/2_) of *Ch*CODHs were 24.6 h for A559W/V610H, 24.5 h for A559H/V610W, 17.7 h for A559H/V610H, and 17.4 h for A559W/V610W, all higher than the 2.4 h of WT. Additionally, the 24 h‐residual activity was maintained at levels of 800–1400 U·mg^−1^. These results align with the reduced oxygen sensitivity observed in the double mutants and suggest that these four variants retain their CO oxidation capability in air. Our findings provide insights into how specific tunnel residue mutations can modulate the enzyme characteristics and oxygen sensitivity of *Ch*CODH variants, offering guidance for gas substrate delivery and practical enzyme catalyst development.

### Structural Analysis of O_2_‐Tolerant *Ch*CODH2 Variants

**Figure 3:**
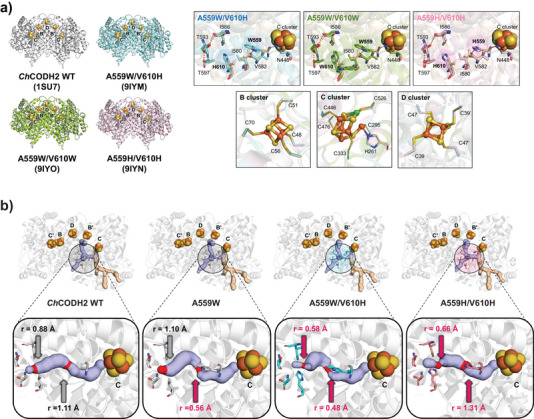
Structural analysis of O_2_‐tolerant *Ch*CODH2 variants. a) Tunnel‐forming residues of *Ch*CODH2 variants and their metal clusters. Structures of *Ch*CODH2 WT (PDB 1SU7, gray) and variants A559W/V610H (PDB 9IYM, blue), A559W/V610W (PDB 9IYO, green), A559H/V610H (PDB 9IYN, pink) are shown. The coordinate residues at the tunnel entrances and clusters of WT and each variant are superimposed (black box). Dotted lines denote hydrogen bonds. Fe (orange spheres), S (yellow spheres), and Ni (green spheres) are depicted. Fe4 displays dual conformations in the C‐cluster, supported by anomalous density. b) Gas tunnels of *Ch*CODH2 WT and variants. The gas tunnels are indicated as non‐selective tunnels (light blue color) and selective tunnels (light orange color). The internal tunnels and bottleneck points (highlighted in red) of WT, A559W, A559W/V610H, and A559H/V610H are shown with the radius (*r*) expanded in black boxes.

To investigate the reason for the improved O_2_ tolerance of the *Ch*CODH2 variants, we determined the crystal structures of the A559W/V610H (Protein Data Bank (PDB) 9IYM), A559H/V610H (PDB 9IYN), and A559W/V610W (PDB 9IYO) variants at a resolution of 1.8–2.1 Å (Figure 3a and Table S6). The overall structures of these variants showed no significant structural differences compared to the known structure of *Ch*CODH2 WT (PDB 1SU7),^[^ ^23^ ^]^ as shown in Figures 3a and S6 (root mean square deviation (RMSD) values: PDB 9IYM 0.319 Å, PDB 9IYN 0.323 Å, and PDB 9IYO 0.308 Å for the 633 Cα atoms). Meanwhile, the structural integrity of the FeS clusters in CODH, which are highly sensitive to oxygen but play a crucial role in CO conversion activity,^[^ ^15^, ^32^, ^33^ ^]^ was also maintained. Furthermore, we demonstrated that the FeS clusters are intact through the *F_o_*‐*F_c_* omit maps (*F_o_*, experimentally measured amplitude; *F_c_*, model‐based amplitude) and Fe anomalous difference maps (Table S7 and Figure S7).

On the other hand, the local environments around the A559 and V610 mutation sites showed some changes compared to those of the *Ch*CODH2 WT (Figure 3a). The I580 side chain was displaced by introducing a bulkier residue (W559 or H559) instead of alanine, with N448 and V582 forming hydrogen bonds with these residues, as reported previously.^[^ ^8^ ^]^ The V610H mutation maintained structural integrity by not disrupting neighboring residues. In the case of the A559W/V610W structure, unexpectedly, two alternative conformations of the V610W side chain were observed, attributed to the inherent flexibility of the residue at the protein surface.

Based on the structures of the *Ch*CODH2 variants, we analyzed changes in the substrate tunnels using the CAVER program^[^ ^34^ ^]^ (Figure 3b). In the WT, the bottleneck of tunnel #2, leading to the active site C‐cluster, is located at the V610 site with a radius of 0.88 Å. However, in the A559W structure (PDB 7XDM), tunnel engineering shifted the bottleneck to position 559 with a radius of 0.56 Å, a shared point of the non‐selective tunnels. For the A559W/V610H variant, while the radius at position 559 (∼0.48 Å) showed minimal change, the radius at position 610, corresponding to the WT bottleneck, was reduced to 0.58 Å. Similarly, in the A559H/V610H variant, the radius at position 559 was 1.31 Å, akin to the original A559H tunnel structure (∼1.43 Å), and the radius at position 610 was 0.66 Å, closely matching that of A559W/V610H. Regrettably, the tunnel for A559W/V610W was excluded from the analysis due to the unclear specification of the tunnel entrance radius, which was caused by the presence of two forms of V610W (Figure S8). Consequently, these findings imply that strategically relocating and constricting bottlenecks in *Ch*CODH2 variants can markedly affect oxygen sensitivity and enzyme performance by redirecting substrate and oxygen flow while maintaining structural integrity.

### Air‐Exposure Structures of *Ch*CODH2 Variants

**Figure 4:**
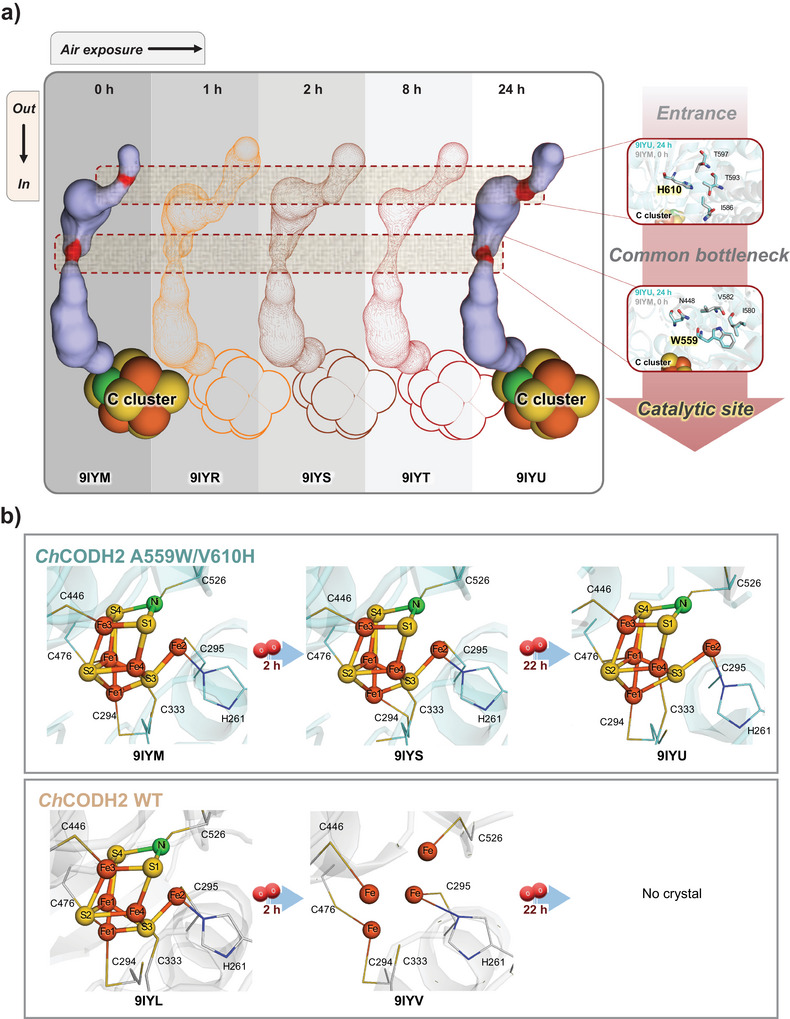
Structural comparisons of the gas tunnel and C‐cluster of air‐exposed *Ch*CODH2 WT and the A559W/V610H variant. a) Tunnel structure changes of *Ch*CODH2 A559W/V610H over varying air exposure time: no exposure, PDB 9IYM; 1 h exposure, PDB 9IYR; 2 h exposure, PDB 9IYS; 8 h exposure, PDB 9IYT; 24 h exposure, PDB 9IYU. The bottleneck points inside the tunnel are highlighted in red. The coordinate residues at the tunnel entrances and common bottleneck are superimposed for 0 h (cyan sticks) and 24 h (gray sticks) structures (burgundy box). The mutated residues W559 and H610 are highlighted in yellow. b) Structural changes in the catalytic site (C‐cluster) of WT and A559W/V610H over varying air exposure time. Different colors were used to distinguish atoms: orange for Fe; yellow for S; green for Ni.

To further explore the relationship between oxygen tolerances and structural changes over time under atmospheric conditions, we obtained snapshots of protein structures at various air exposure times (Figure 4 and Table S6). Among the three *Ch*CODH2 variants (A559W/V610H, A559H/V610H, and A559W/V610W), we selected the variant with the best air stability, A559W/V610H, to compare with *Ch*CODH2 WT for the crystallographic time‐course experiment. To solve the air‐exposed structures, highly purified proteins of *Ch*CODH2 WT and A559W/V610H variant were directly exposed to air for varying times (0–24 h), followed by crystallization under anaerobic conditions. As a result, we successfully solved the crystal structures of the O_2_‐tolerant A559W/V610H variant at various air exposure times (0, 1, 2, 8, and 24 h) (Figure 4). In contrast, protein crystals for the WT were only obtained from samples exposed for 0–2 h. When we conducted tunnel analysis and compared the A559W/V610H structures over time (Figures 4a and S9), the residues at the two bottleneck positions showed minimal differences, and the overall tunnel structure remained largely unchanged, suggesting that the enhanced oxygen tolerance is correlated with the structural stability of the enzyme under oxygenated conditions.

Given that FeS metal clusters are generally known to be sensitive to oxygen exposure^[^ ^2^, ^35^, ^36^ ^]^ and play a crucial role in the enzymatic reaction and electron transfer of CODH, we tracked changes in the FeS metal clusters B, C, and D in both WT and A559W/V610H structures with varying air exposure times (Figures 4b and S10). To monitor the structural integrity of these clusters, anomalous difference Fourier maps for Fe, S, and Ni atoms were analyzed (Tables S7 and S8; Figure S11). After 2 h of air exposure, the B‐cluster remained relatively intact in both *Ch*CODH2 WT and A559W/V610H variant, corroborating previous observations of oxygen vulnerability and decay.^[^ ^37^ ^]^ On the other hand, the electron density and omit maps for Ni, as well as most Fe sites, in the C‐ and D‐clusters of *Ch*CODH2 WT were absent or severely diminished, indicating partial cluster collapse upon direct air exposure (Figure S10). Nonetheless, residual anomalous signals for Fe at the C‐cluster (Figures S10 and S11) suggest that the cluster was not entirely lost. Although the D‐cluster is located near the protein surface and not directly connected to the substrate tunnels, it was degraded in the WT but remained intact in the A559W/V610H variant after 24 h of air exposure. This striking difference may suggest that tunnel constriction protects the C‐cluster from oxidative damage and may indirectly help preserve the structural integrity of spatially distant clusters like the D‐cluster by maintaining redox balance or other contributing factors. Furthermore, the FeS clusters in the A559W/V610H variant remained intact even after 24 h of air exposure, fitting precisely into the omit and Fe and Ni anomalous maps (Figure S11). These results suggest that while the *Ch*CODH2 WT becomes structurally unstable upon air exposure, the A559W/V610H variant with a narrowed bottleneck maintains its structure and exhibits lower oxygen sensitivity. This implies that the C‐cluster collapse may serve as the primary trigger for overall enzyme destabilization. When oxygen reaches the catalytic C‐cluster, it destabilizes the NiFeS cluster, leading to overall protein instability. Loss of Ni/Fe signals in the C‐cluster of air‐exposed *Ch*CODH2 WT (Figures S10 and S11), along with reduced enzymatic activity, indicates catalytic center disruption, aligning with O_2_‐induced cleavage of metal–sulfur bonds in NiFeS clusters.^[^ ^37^ ^]^ Thus, the structural collapse of the C‐cluster in *Ch*CODH2 WT is identified as a key event of oxygen sensitivity, ultimately resulting in enzyme inactivation. In this context, the C‐cluster can be effectively secured by the narrowed bottleneck in the A559W/V610H variant.

### Electrochemical CO Oxidation of O_2_‐Tolerant CODH Variants in Air

**Figure 5:**
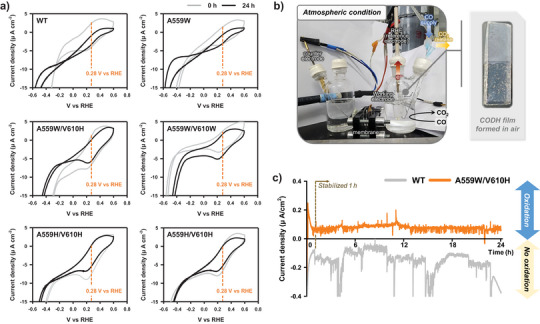
Electrochemical reactions of O_2_‐tolerant *Ch*CODH2 variants in air. a) Viologen‐free CO oxidation of air‐exposed *Ch*CODH2 film by cyclic voltammetry (CV). CV was performed on fluorine‐tin oxide (FTO) electrodes with CODH films after 24 h air exposure under 100% (*v*/*v*) CO, 25 °C, and pH 8. Gray lines represent no exposure, and black lines represent 24 h exposure. The observed potential (V versus reversible hydrogen electrode (RHE)) in CO oxidation reactions is indicated by an orange dotted line at 0.28 V versus RHE. All measurements were conducted in triplicate to verify reproducible CV profiles. b) Setup for long‐term electrochemical CO oxidation under atmospheric conditions. The reaction was performed at room temperature using an electrochemical cell comprising an anode, cathode, and electrolyte with real‐time CO gas injection (blue highlights) to observe current changes during CO oxidation to CO_2_ (yellow highlights). The enlarged CODH film was prepared under air exposure. See the Supporting Information experimental procedures section for details. c) 24 h electrochemical CO oxidation of *Ch*CODH2 A559W/V610H. The current of WT (gray lines) and A559W/V610H (orange lines) was compared in electrochemical reactions under atmospheric conditions. The dashed line indicates the baseline of zero current density.

To compare the changes in reaction potential due to oxygen exposure in *Ch*CODH2 WT and engineered enzymes, we conducted electrochemical CO oxidation reactions using protein films (Figure 5a). Oxygen present during the CODH‐mediated CO oxidation can easily react with reduced electron mediators such as viologens, so using mediator‐free electrochemical reactions helps to exclude this influence. Protein films of *Ch*CODH2 WT and variant proteins were prepared based on their air exposure times (0–24 h) (Figure S12). Electrochemical reactions of the air‐exposed WT and variants showed that the WT enzyme exposed to oxygen for 24 h exhibited a notable decrease in current compared to the enzyme at 0 hour (0.28 V versus RHE). The single mutant A559W displayed less current change compared to the WT. As expected, the four double mutants (A559H/V610H, A559 H/V610W, A559W/V610H, and A559W/V610W) showed no significant shifts in redox potential. The measured current was proportional to their enhanced oxygen tolerance, in line with the principle that current reflects enzyme activity and loading. Furthermore, CODH activity measured by current is a highly oxygen‐sensitive electrochemical reaction, as observed by both the Dobbek^[^ ^15^ ^]^ and Fourmond^[^ ^33^ ^]^ groups. This indicates that the current generated by CODH enzymes is limited by their oxygen sensitivity, and our engineered variants demonstrate greater robustness under oxygenated conditions.

To further investigate, we extended the electrochemical reaction time and conducted 24 h electrochemical CO oxidation reactions (Figure 5b,c). The electrochemical cell and setup were arranged under ambient conditions to allow oxygen exposure, and current changes during the enzyme reaction were monitored in real‐time (Figure 5b). Through this long‐term operation over 24 h, the variant A559W/V610H maintained its electrochemical CO oxidation activity well, retaining 81% of the stabilized current at the 1 h mark, after 24 h (Figure 5c). The current at 1 h was used as a baseline due to the stabilization of the current by that time. In contrast, the WT enzyme exhibited negative current values, likely due to its inactivation from oxygen exposure leading to non‐specific background currents. This suggests that no CO oxidation activity occurred for the WT, indicating the effectiveness of our engineered variants in maintaining activity under these conditions. This demonstrates that our engineered CODHs, particularly the double mutants, maintain stable electrochemical CO oxidation activity under prolonged oxygen exposure, suggesting their potential for practical applications where oxygen presence is unavoidable.

## Conclusion

This study provides valuable insights into enzyme engineering, particularly the role of O_2_ invasive tunnel modifications in improving oxygen sensitivity through preserving the FeS clusters. The strategic creation of additional bottlenecks to block oxygen entry without affecting substrate flow presents a novel approach to enzyme stabilization under oxidative conditions. This method can be applied to other oxygen‐sensitive enzymes, such as [FeFe]‐hydrogenases,^[^ ^38^, ^39^, ^40^ ^]^ potentially broadening its utility across various biocatalytic processes. Furthermore, our findings suggest that oxygen sensitivity and catalytic efficiency are not mutually exclusive. By carefully selecting mutation sites and understanding their structural impacts, it is possible to engineer enzymes that resist oxidative damage while maintaining high catalytic activity. This challenges the conventional trade‐off assumption between stability and activity in enzyme engineering. The ability to maintain enzyme activity under prolonged air exposure opens up new possibilities for industrial applications, particularly in processes involving waste gases and syngases that contain oxygen. The air‐viable, fast CODHs by protein engineering could be used in bioconversion processes, gas cleaning, and even in the development of biosensors and bioelectrochemical systems where oxygen exposure is a concern.

In conclusion, our study demonstrates the potential of tunnel engineering in creating robust, oxygen‐tolerant biocatalysts. The insights gained from this research not only advance the field of enzyme engineering but also pave the way for the practical application of these enzymes in challenging industrial environments. Further research into the exact mechanisms of oxygen interaction with O_2_‐sensitive metallo‐cofactors will continue to refine and enhance the design of oxygen‐stable enzymes.

## Supporting Information

The Supporting Information includes experimental procedures, Tables S1–S8, and Figures S1–S15, and is provided as a PDF file. References^[^ ^41^, ^42^, ^43^, ^44^, ^45^, ^46^, ^47^, ^48^, ^49^, ^50^, ^51^, ^52^, ^53^, ^54^, ^55^ ^]^ cited therein are listed in the main reference list.

## Author Contributions

Y.H.K. and S.M.K. conceived and planned all of the experiments. S.M.K. performed the bioinformatic analysis and gene cloning. S.M.K. and J.K. performed the biochemical characterization, kinetic analysis, and feasibility evaluation, all under the supervision of Y.H.K. S.M.K. and S.H.K. engineered the gas tunnels and performed their structural analysis. J.R. and H.K. conducted the electrochemical analysis. Y.H.K. and S.M.K. wrote the manuscript. K.S.Y. and H.H.L. conceived and carried out the structural work and wrote the structural sections of the manuscript. H.Y. and J.S.J. assisted in the structure determination. Y.H.K., H.H.L., and S.M.K. reviewed the manuscript.

## Conflict of Interests

The authors declare no conflict of interest.

## Supporting information



Supporting Information

## Data Availability

The datasets generated and analyzed during the current study are available from the corresponding author upon request. The kinetic data in this study are provided in the Supplementary Information. The atomic coordinates and structure factors for the *Ch*CODH2 variants have been deposited in the Protein Data Bank (http://www.rcsb.org) under the accession codes 9IYL, 9IYM, 9IYN, 9IYO, 9IYR, 9IYS, 9IYT, 9IYU, and 9IYV.
