# Supplementary material for: Fortification of FeS Clusters Reshapes Anaerobic CO Dehydrogenase into an Air‐Viable Enzyme Through Multilayered Sealing of O2 Tunnels
Source: Angew Chem Int Ed Engl. 2025 Jun 12;64(33):e202508565. doi: 10.1002/anie.202508565 (PMC12338426; doi:10.1002/anie.202508565)
Supplement: Supplementary file 1 — Supporting Information [file ANIE-64-e202508565-s001.docx]

**Supporting Information**

**Fortification of FeS Clusters Reshapes Anaerobic CO Dehydrogenase into an Air-Viable Enzyme through Multilayered Sealing of O_2_ Tunnels**

**[Table of contents]**

**Experimental procedures**

*Ch*CODH2 gene synthesis and cloning 3

Protein expression and purification 3

Size-exclusion chromatography with multiangle light scattering (SEC-MALS) 4

Activity measurement 4

Kinetic analysis 4

Air stability of immobilized *Ch*CODHs 5

Crystallization and structural determination 5

X-ray absorption spectroscopy and anomalous difference map 6

Electrochemical CO oxidation of CODHs 6

Bioinformatics analysis 7

**Tables S1‒S8**

Table S1 | Key diffusion-limited enzymes 9

Table S2 | The list of primers used in this study 10

Table S3 | Summary of O_2_ sensitivity in all tested variants 11

Table S4 | Predicted stability change of *Ch*CODH2 variants 12

Table S5 | Nickel content and kinetic parameters of *Ch*CODH2 variants normalized to Ni content 13

Table S6 | Data collection and structure refinement of *Ch*CODH2 WT and variants 14

Table S7 | Anomalous data collection 16

Table S8 | S anomalous data collection 17

**Figures S1‒S15**

Figure S1 | Water molecules and coordinating residues in the non-selective tunnels of *Ch*CODH2 WT and variants 18

Figure S2 | Sequence alignment of *Ch*CODH-related proteins 19

Figure S3 | Specific activity of *Ch*CODH WT and variants at varying oxygen concentrations 20

Figure S4 | Workflow for evaluating air stability of Ni-NTA immobilized *Ch*CODH2 variants 21

Figure S5 | Residual activity and half-life time (*t*_1/2_) of *Ch*CODHs under atmospheric conditions 22

Figure S6 | Structural differences of *Ch*CODH2 WT and variants 23

Figure S7 | The *Fo-Fc* omit map and Fe anomalous maps for the B, C, and D-clusters in *Ch*CODH2 WT and variants 24

Figure S8 | Gas tunnels of *Ch*CODH2 A559W/V610W 25

Figure S9 | Changes in the tunnel radius of air-exposed *Ch*CODH2 A559W/V610H 26

Figure S10 | Omit maps (Fo–Fc) for B, C, and D-clusters in *Ch*CODH2 WT and A559W/V610H variant over air exposure time 27

Figure S11 | Fe and Ni anomalous difference Fourier maps after time dependent air exposure 28

Figure S12 | Electrode validation and electrochemical air stability of *Ch*CODH2 variants 29

Figure S13 | SEC-MALS analysis of *Ch*CODH2 A559W/V610H oligomeric state 30

Figure S14 | Nonlinear hyperbolic kinetic profiles across *Ch*CODH2 variants 31

Figure S15 | Determination of half-maximal inhibitory oxygen concentration (IC_50_) in *Ch*CODH2 and variants 32

**Experimental procedures**

***Ch*CODH2 gene synthesis and cloning**

The *Ch*CODH2 gene (CHY_0085)^[5b]^ was codon-optimized and synthesized with *Nde*I and *Bam*HI restriction sites, followed by sequence verification by Macrogen (Korea) using the GenBank sequence (accession no. NC_007503). The synthesized gene fragments were then inserted into the pET28a vector (Novagen), which includes a His-tag and a thrombin cleavage site. For site-directed mutagenesis, the QuikChange method from Stratagene was used with Pfu DNA polymerase. After PCR, the products were treated with *Dpn*I (2 U·µL^‒1^) at 37°C for 60 minutes to digest the parental plasmid. The resulting plasmids were purified and transformed into *E. coli* DH5α cells. Point mutations were confirmed by DNA sequencing. The verified pET28a-*Ch*CODH2 plasmids and their variants were subsequently introduced into *E. coli* BL21(DE3) harboring pRKISC^[28]^. Details on primers, vectors, and strains are summarized in Table S2.

**Protein expression and purification**

Cultures were grown in a modified Terrific Broth medium with supplements including 0.02 mM NiCl_2_, 0.1 mM FeSO_4_, 2 mM L-cysteine, kanamycin (50 µg·mL^‒1^), and tetracycline (10 µg·mL^‒1^) at 37°C. At an OD600 of 0.4–0.6, expression of recombinant CODHs and their variants was initiated by adding 0.2 mM IPTG, along with 0.5 mM NiCl_2_, 1 mM FeSO_4_, and 50 mM KNO_3_ after purging with nitrogen. The induction was carried out overnight at 30°C, and cells were subsequently harvested and stored at -70°C under aerobic conditions. Purification steps were carried out in an anaerobic glove box (model B, COY Laboratory Products Inc., Michigan, USA) under a 95% N_2_/5% H_2_ atmosphere at room temperature. Anaerobic sonication on ice was used for cell lysis. Centrifuged lysates were purified using Ni-NTA affinity chromatography (Qiagen) with a buffer containing 2 mM dithioerythritol (DTE) and 2 µM resazurin, except during washing and elution steps for O_2_ inactivation assays where these components were omitted. Protein concentrations were measured with the Bradford assay. SDS-PAGE analysis on 12% gels showed single bands at approximately 69 kDa, consistent with the calculated sizes of His₆-tagged proteins (67 kDa + 2 kDa His6-tag). Western blotting with anti-His-tag antibodies confirmed the presence and integrity of His-tagged proteins.

For crystallization under anaerobic conditions, additional purification of *Ch*CODH2 WT and variants (A559H/V610H, A559W/V610H, and A559W/V610W) was performed using size-exclusion chromatography (HiLoad 16/600 Superdex 200 prep grade, GE Healthcare Bio-Sciences, PA, USA) in the anaerobic glove box. The column was equilibrated with a buffer containing 20 mM Tris-HCl (pH 7.5) and 3 mM dithiothreitol (DTT). For air-exposed CODHs, purification using Ni-NTA resin was done similarly but under aerobic conditions. Further size-exclusion chromatography was also performed aerobically using a buffer containing 20 mM Tris-HCl (pH 7.5) and 3 mM DTT.

**Size-exclusion chromatography with multiangle light scattering (SEC-MALS)**

Size-exclusion chromatography with multiangle light scattering detection on the *Ch*CODH2 A559W/V610H variant was performed aerobically using a Wyatt MiniDAWN TREOS MALS instrument and a Wyatt Optilab rEX differential refractometer, in-line with a Superdex 200 10/300 GL analytical column (GE Healthcare). The gel-filtration column was pre-equilibrated with a buffer containing 20 mM Tris-HCl (pH 7.5) and 3 mM DTT and normalized using bovine serum albumin (Sigma‒Aldrich). Prior to measurement, the sample was centrifuged at 11,000 ×g for 10 min at 4°C. 100 µL of 4 mg·mL^‒1^ protein sample was injected, and elution was performed at a flow rate of 0.4 mL·min^‒1^. Data were analysed using the Zimm model for static light-scattering data fitting and graphed using an EASI graph with an ultraviolet peak in ASTRA V software (Wyatt), as shown in Figure S13.

**Activity measurement**

CO oxidation activity was measured at 30°C for practical applications^[18a]^, benefiting from spontaneous reactions and easy coupling with mesophilic enzymes. The activity was assessed using screw-cap cuvettes with CO headspace, saturated with CO. The CO-dependent reduction of oxidized ethyl viologen (EV_ox_, ε_578_ = 10,000 M^-1^ × cm^-1^)^[29]^ was monitored. In each screw-cap cuvette, 2 mL of reaction buffer containing 20 mM EV_ox_, 0–250 µM O_2_, and 50 mM HEPES/NaOH buffer (pH 8) saturated with CO (prepared by flushing 50 mL of stock solution with CO gas for 1 hour)^[30]^ was used. Reactions were initiated by injecting 0.1–0.2 µg of enzyme. Trace amounts of oxygen during enzyme preparation may slightly affect CODH activity due to the absence of a reducing agent (DTE), resulting in minor differences in specific activity measurements. To evaluate the effect of O_2_ inactivation, we measured the residual activity of the enzyme at various O_2_ concentrations^[5b]^. Prior to assaying, CODHs (2–4 µg) were incubated in 50 mM HEPES (final volume of 200 µL) for 2 minutes with added O_2_. The reaction mixture was then diluted 200–400 times to achieve a final O_2_ concentration below 0.6 µM. One unit of CODH activity was defined as the amount of enzyme required to reduce 1 µmol of EV_ox_ per minute at 30°C and pH 8.

To analyze the Ni content, we cultured the cells following our standard protocol. To prevent non-specific binding of Ni during purification, we used Co-NTA resin instead of Ni-NTA. Size-exclusion chromatography (SEC) was subsequently performed using an ÄKTA Pure FPLC system (Cytiva), followed by ICP-OES analysis. We normalized the activity and turnover number by dividing the Ni content.

**Kinetic analysis**

To determine the kinetic parameters for EV_ox_, a CO-saturated buffer (0.91 mM CO, 99.998% as per Henry’s law) was used at 30°C. A rubber septum-stopped cuvette containing 0.5–8 mM EV in 50 mM HEPES/NaOH buffer (pH 8) had its headspace purged with CO for 1 hour. The reaction was initiated by injecting 0.1–0.2 µg of recombinant CODHs to monitor the initial velocity. One unit of CODH activity was defined as the amount of enzyme that reduces 1 μmol of viologen per minute per mg of protein. For the kinetic parameters towards CO, two CO-saturated buffers were prepared: one with 0.91 mM CO (99.998% per Henry’s law) and another with 45.5 µM CO (5% CO/95% N_2_ *v*/*v* per Henry’s law), both maintained at 30°C. Reaction mixtures containing final CO concentrations of 10, 20, 40, 80, and 160 µM were prepared by diluting these buffers with 50 mM HEPES pH 8 buffer to achieve a final concentration of 20 mM EV. The enzyme reaction was initiated by injecting the prepared reaction buffers into the enzyme (0.1–0.2 µg). The change in absorbance at 578 nm was monitored spectrophotometrically at 30°C in an anoxic glove box. Kinetic parameters (*k*_cat_ and *K*_m_) for CO and EV_ox_ were calculated using nonlinear hyperbolic regression (Figure S14). All enzymatic activities were determined in triplicate. IC_10_, IC_50_, and IC_90_ values were calculated through nonlinear regression (four-parameter logistic curve, Figure S15).

**Air stability of immobilized *Ch*CODHs**

To assess the air stability of *Ch*CODH2 variants under atmospheric conditions, we prepared Ni-NTA immobilized enzymes and measured residual CO oxidation activity over time following oxygen exposure. Enzyme immobilization was performed in an anaerobic chamber using 0.5 mL of Ni-NTA agarose resin and 10 mg of purified His6-tagged enzyme, incubated at room temperature for 2 hours in 50 mM sodium phosphate buffer (pH 8). The immobilized enzymes were then washed twice with CO-purged 50 mM sodium phosphate buffer (pH 8) to ensure an oxygen-free environment and stored in the chamber. For the air exposure tests, the immobilized enzymes were exposed to ambient air at room temperature for various durations up to 24 hours. At each time point, samples were collected under anaerobic conditions using disposable columns. The samples were washed twice with CO-purged 50 mM sodium phosphate buffer (pH 8) to remove any residual oxygen. The oxygen-free immobilized enzymes were then eluted with elution buffer (50 mM NaH_2_PO_4_, 30 mM NaCl, 250 mM imidazole, pH 8) to obtain the free enzyme. The protein concentration was determined, and the residual CO oxidation activity was measured.

**Crystallization and structural determination**

To solve the crystal structures of the *Ch*CODH2 A559W/V610H, A559H/V610H, and A559W/V610W variants (PDB IDs: 9IYM, 9IYN, 9IYO), crystals were developed using the hanging drop vapor diffusion method. This involved mixing 2 µL of protein solution (10 mg/mL) with 2 µL of reservoir solution containing 0.1 M HEPES/NaOH (pH 7.0), 0.2 M MgCl_2_, and 25% (*w*/*v*) polyethylene glycol 3,350. For cryoprotection, the crystals were immersed in a reservoir solution supplemented with 10% (*w*/*v*) glycerol. Data collection was conducted at 100 K with 1° oscillations on the 5C beamlines at Pohang Light Source (PLS) and SPring8 (SP8), using a wavelength of 1.0000 Å. The crystals diffracted between 1.28 Å and 2.50 Å, and the data were processed using the HKL2000^[31]^ and XDS^[32]^ software packages. Structural determination employed the molecular replacement method, using the *Ch*CODH2 WT model (PDB ID: 1SU7) ^[10]^ as a reference. Manual model building was performed in *Coot*^[33]^, followed by refinement with *PHENIX* (ver. 1.20.1_4487)^[34]^ and *CCP4* refmac5^[35]^, excluding hydrogen atoms. The refinement involved several cycles of model building, simulated annealing, and *B*-factor refinement. The final atomic coordinates and structural factors were submitted to the Protein Data Bank under codes 9IYL, 9IYM, 9IYN, and 9IYO. Detailed refinement statistics are provided in Table S6.

To assess the impact of oxygen exposure on protein structure, purified samples of *Ch*CODH2 WT and the A559W/V610H variant were subjected to aerobic conditions for up to 24 hours. Using the hanging drop method, 2 µL of an 8 mg/mL protein solution was combined with 2 µL of reservoir solution (0.1 M HEPES/NaOH pH 7.0, 0.2 M MgCl_2_, and 25% (*w*/*v*) polyethylene glycol 3,350). The crystals were cryoprotected with 12.5% (*w*/*v*) glycerol in the reservoir solution. Data were collected at 100 K in 1° oscillations using the same beamlines and conditions as the anaerobic samples. The structural analysis procedures mirrored those used for the anaerobic variants, ensuring consistency in methodology. The resulting atomic coordinates and structural factors are archived in the Protein Data Bank (PDB codes: 9IYR, 9IYS, 9IYT, 9IYU, and 9IYV). All structural figures were generated using PyMOL (Schrödinger, LLC; https://www.pymol.org).

**X-ray absorption spectroscopy and anomalous difference map**

Fluorescence scans on *Ch*CODH2 variant crystals were conducted at the 5C beamlines of the Pohang Light Source (PLS) and SPring8 (SP8), focusing on the X-ray absorption edges of iron and nickel ions. Anomalous diffraction data were collected at the peak absorption edges for iron (7.140 keV) and nickel (8.374 keV for anaerobic A559W/V610H and 8.348 keV for all other samples). These data sets were processed and scaled using the *HKL2000* software package^[36]^. Anomalous data-collection statistics are detailed in Table S7 and S8. Anomalous difference Fourier map calculations were performed using the fast Fourier transform (FFT) in the *CCP4* Refmac5 program^[35]^.

**Electrochemical CO oxidation of CODHs**

The electrochemical performance of *Ch*CODH2 WT and its variants was assessed using cyclic voltammetry (CV) with an SP-150 Biologic potentiostat (BioLogic Science Instruments, France). Measurements were conducted in an aerobic custom-built cell featuring a working electrode coated with CODH enzyme films^[9]^, a platinum wire counter electrode, and an Ag/AgCl reference electrode. To prepare stable enzyme films, 2 µL of a 1:3 mixture of CODH and polymyxin was applied to the electrode, following established protocols^[9, 37]^. For the electrode preparation, a mixture of 5 µL of 400 mM carbodiimide and 5 µL of 100 mM *N*-hydroxysulfosuccinimide was applied to the fluorine-doped tin oxide (FTO) electrode surface. After a 20-minute incubation, 10 µL of the *Ch*CODH2 solution (containing 10 µg of protein) was added. The electrode was dried under anaerobic or aerobic conditions as required by the experiment. For aerobic conditions, exposure times were set at 0, 2, 4, 6, or 24 hours at room temperature.

The electrolyte solution consisted of a high-concentration 50 mM HEPES/NaOH buffer (pH 8.0), purged with pure CO gas for at least 1 hour before the reaction. The prepared electrode was placed in a 3 mL cell containing the same buffer, with 2 mM DTE and 2 µM resazurin added. CV was performed at a scan rate of 10 mV s^−1^, within a potential range of ‒0.5 V to 0.5 V versus the reversible hydrogen electrode (RHE). All potentials were referenced to the Ag/AgCl electrode and adjusted to the RHE standard using the Nernst equation^[38]^: *E* (V vs. RHE) = *E* (V vs. Ag/AgCl) + 0.0592 × *pH* + 0.197. Each test was conducted in triplicate for accuracy and reproducibility. To evaluate the long-term electrochemical response under oxygen exposure, protein films of *Ch*CODH2 WT and A559W/V610H were prepared and measured in an oxygen environment. The experimental conditions mirrored those of the previous CV experiment with the following exceptions: the potential was applied at 0.28 V vs. RHE (the observed potential for CO oxidation), and the protein films were dried aerobically for 24 hours. The experiment lasted for 24 hours with CO gas supplied continuously.

To confirm that the observed CV results were not due to differences in enzyme-electrode affinity, additional control experiments were performed. Cyclic voltammetry (CV) was conducted using only the FTO electrode, and separately using the FTO electrode supplemented with the same concentration of carbodiimide and *N*-hydroxysulfosuccinimide.

**Bioinformatics analysis**

Amino acid sequence homologues were identified using BLAST, and multiple sequence alignments were performed with ClustalW. Hybrid-cluster proteins lacking CODH activity were excluded from the analysis. Conserved domains (CDs) and clusters of orthologous groups (COGs) of proteins were analysed using the CD-Search tool available at the National Center for Biotechnology Information (NCBI; http://www.ncbi.nlm.nih.gov). For substrate tunnel analyses, we utilized CAVER3.0 software^[24]^ (http://www.caver.cz). Icons in Fig. 2d were created by Freepik from Flaticon (www.flaticon.com).

**Tables S1‒S8 and Figures S1‒S15**

**Table S1 | Key diffusion-limited enzymes.**

| **Substrate diffusion rate* (s**^‒1^**·M**^‒1^**)** | **Diffusion-limited enzyme†** | **Reference** |
| --- | --- | --- |
| 10^7^ ‒ 10^8^ | *Acetylcholin esterase* | Source^[8b]^ |
|  | *Carbonic anhydrase* | Source^[8b]^ |
|  | *β-lactamase* | Source^[39]^ |
|  | *Catalase* | Source^[40]^ |
| 10^8^ ‒ 10^9^ | ***CODH*** | Source^[8a]^ |
|  | *Cytochrome c peroxidase* | Source^[8b]^ |
|  | *Triosephosphate isomerase* | Source^[8b]^ |
|  | *Fumarase* | Source^[41]^ |
| > 10^9^ | *Superoxide dismutase* | Source^[8b]^ |

The table lists major enzymes that are limited by substrate diffusion rates, categorized by their substrate diffusion rates (in s^‒1^·M^‒1^). These enzymes are crucial for various biological processes, and their efficiency is often constrained by how quickly their substrates can diffuse. CODH is highlighted in magenta.

* The rate at which substrates diffuse to the enzyme.

† The enzyme whose activity is limited by the rate of substrate diffusion.

**Table S2 | The list of primers used in this study.**

| **Mutation** | **Primer sequence (5' to 3')** | | **Vector** | **Host*** |
| --- | --- | --- | --- | --- |
|  | **forward primer** | **reverse primer** |  |  |
| Ch2_E43H | cggtttcggccacccggtctgtgctgccgtc | acagaccggtgtggccgaaaccgcactgcgg | pET28a | *E. coli* BL21(DE3) |
| Ch2_Q206H | gcggatgcgcacaacctgctgctgggtg | cagcaggttgtgcgcatccgcgtcgcaa |  |  |
| Ch2_K450H | caacaacgttcacgtgccgcaggaccaaaac | tcctgcggcacgtgaacgttgttgcaacc |  |  |
| Ch2_L583H | cattggtgtgcacccgccgatcaccggca | gatcggcgggtgcacaccaatgtgggtcgg |  |  |
| Ch2_I586H | ctgccgccgcacaccggcagcct | ctgccggtgtgcggcggcagcacaccaatg |  |  |
| Ch2_T593H | agcctgccggtgcaccaaatcctgaccagcag | tcaggatttggtgcaccggcaggctgccggtgat |  |  |
| Ch2_T597H | cccaaatcctgcacagcagcgttaaagatattac | tttaacgctgctgtgcaggatttgggtcaccggca |  |  |
| Ch2_S599H | cctgaccagccacgttaaagatattaccgg | tatctttaacgtggctggtcaggatttggg |  |  |
| Ch2_I603H | cgttaaagatcacaccggtggctacttcat | agccaccggtgtgatctttaacgctgctgg |  |  |
| Ch2_V610A | gctacttcatcgcggaactggacccggagacc | ggtccagttccgcgatgaagtagccaccgg |  |  |
| Ch2_V610H | gctacttcatccacgaactggacccggagacc | ggtccagttcgtggatgaagtagccaccgg |  |  |
| Ch2_V610W | gctacttcatctgggaactggacccggagacc | ggtccagttcccagatgaagtagccaccgg |  |  |
| Ch2_A559H | cgcggcggaacacatgcatgagaaggcggtgg | tctcatgcatgtgttccgccgcgctcgcaacc |  |  |
| Ch2_A559W | gcgcggcggaatggatgcatgagaaggcggtgg | tctcatgcatccattccgccgcgctcgcaacc |  |  |

* The host *E. coli* BL21(DE3) strain contains the pRKISC plasmid^[27]^ carrying Fe–S cluster assembly proteins.

**Table S3 | Summary of O_2_ sensitivity in all tested variants.**

| **Group** | **Related residues** | **Specific activity***  **(U·mg^-1^)** | **Inhibitory oxygen concentration (IC, µM)** | | |
| --- | --- | --- | --- | --- | --- |
|  |  |  | **IC_10_†** | **IC_50_†** | **IC_90_†** |
| ***Ch*CODH2** | A559/V565/I580‡ | 900 ± 28.2 | 0.5 ± 0.1 | 0.6 ± 0.01 | 1.0 ± 0.3 |
| ***Ch*CODH2 variants** | L583H | 180 ± 0.8 | 8.5 ± 1.7 | 10.0 ± 0.6 | 13.0 ± 3.0 |
|  | I586H | 1170 ± 19.1 | 2.3 ± 2.6 | 6.2 ± 2.4 | 25.2 ± 5.2 |
|  | T593H | 1500 ± 61.2 | 3.8 ± 2.8 | 7.8 ± 1.5 | 20.0 ± 1.2 |
|  | T597H | 1260 ± 70.0 | 2.5 ± 3.2 | 6.0 ± 2.0 | 25.5 ± 6.2 |
|  | V610H | 1750 ± 71.4 | 9.3 ± 2.7 | 12.0 ± 0.3 | 16.8 ± 0.9 |
|  | A559H^‡^ | 1900 ± 63.5 | 90.7 ± 8.6 | 92.9 ± 10.3 | 97.1 ± 11.5 |
|  | A559H/V610A | 960 ± 6.1 | 21.5 ± 5.4 | 26.5 ± 1.9 | 32.4 ± 4.4 |
|  | A559H/V610H | 1070 ± 71.4 | 24.5 ± 24.8 | 184.6 ± 67.6 | 481.7 ± 78.1 |
|  | A559H/V610W | 450 ± 12.9 | 10.6 ± 17.1 | 70.8 ± 40.4 | NC |
|  | A559W‡ | 2000 ± 13.2 | 34.9 ± 1.4 | 38.2 ± 1.1 | 48.9 ± 0.2 |
|  | A559W/V610A | 1680 ± 59.4 | 6.1 ± 6.3 | 15.0 ± 6.3 | 58.6 ± 14.8 |
|  | A559W/V610H | 3480 ± 254.2 | 13.8 ± 22.8 | 54.9 ± 36.9 | 851.9 ± 455.7 |
|  | A559W/V610W | 3390 ± 358.6 | 6.1 ± 9.7 | 41.0 ± 19.5 | 451.6 ± 53.0 |

* Enzymatic activities used to assay O_2_ sensitivity are calculated as the mean of three independent measurements. One unit is defined as the 1 μmol reduced viologen·min^‒1^.

† Inhibitory oxygen concentration (IC) values represent the oxygen concentrations that inhibit enzyme activity by 10% (IC_10_), 50% (IC_50_), and 90% (IC_90_). The data represent the mean ± S.D., as determined from *n* = 3 independent experiments. NC indicates values that were not calculated.

‡ The values were derived from the reference^[5b]^.

**Table S4 |** **Predicted stability change of *Ch*CODH2 variants.**

| **Variant (Active)** | **ΔEnergy (REU)*** | **Variant (Inactive)** | **ΔEnergy (REU)*** |
| --- | --- | --- | --- |
| L583H | 0.48 | E43H | –4.48 |
| I586H | –89.64 | K450H | –5.21 |
| T593H | –44.94 | Q206H | 2.67 |
| T597H | –203.00 | S599H | 4.70 |
| V610H | –25.68 | I603H | –4.61 |

Predicted total energy differences (ΔEnergy) of histidine-substituted *Ch*CODH2 variants compared to the WT enzyme (*Ch*CODH2, PDB ID: 1SU7), calculated using the MutationExplorer platform^[41]^ (https://proteinformatics.uni-leipzig.de/mutation_explorer). Negative values indicate predicted stabilization, while positive values suggest destabilization relative to the WT.

* REU: Rosetta Energy Units, as calculated by MutationExplorer. ΔEnergy represents the total energy difference between each mutant and the WT structure (PDB: 1SU7).

**Table S5 | Nickel content and kinetic parameters of *Ch*CODH2 variants normalized to Ni content**

| CODH variant | Substrate | Ni content  (Ni mol/  Enzyme mol) | Specific activity ^a^  (U·mg^-1^) | Normalized  Specific activity ^b^  (U·mg^-1^) | *K*_m_ ^c^  (mM) | *k*_cat_ ^d^  (s^-1^) | *k*_cat_^n e^  (s^-1^) | *k*_cat_/*K*_m_  (s^-1^·mM^-1^) | *k*_cat_^n^/*K*_m_  (s^-1^·mM^-1^) |
| --- | --- | --- | --- | --- | --- | --- | --- | --- | --- |
| *Ch*CODH2 | CO | 0.42 ± 0.07 | 1300 ± 108.5 | 3100 ± 258.9 | 0.020 ± 0.002 | 1500 ± 67 | 3600 ± 160 | 76000 ± 3800 | 179000 ± 19600 |
|  | EV |  | 900 ± 28.2 | 2150 ± 67.3 | 1.8 ± 0.1 | 1000 ± 16 | 2400 ± 38 | 560 ± 15 | 1300 ± 77 |
| A559H | CO | 0.55 ± 0.05 | 2100 ± 118.0 | 3840 ± 216 | 0.061 ± 0.002 | 2300 ± 38 | 4200 ± 69 | 37000 ± 990 | 69000 ± 2500 |
|  | EV |  | 1900 ± 63.5 | 3480 ± 116.3 | 2.1 ± 0.1 | 2300 ± 72 | 4200 ± 130 | 1100 ± 23 | 2000 ± 110 |
| A559W | CO | 0.42 ± 0.02 | 1800 ± 111.0 | 4300 ± 265.5 | 0.034 ± 0.002 | 1800 ± 49 | 4300 ± 120 | 53000 ± 2300 | 126000 ± 8200 |
|  | EV |  | 2000 ± 13.2 | 4780 ± 31.6 | 2.0 ± 0.1 | 2100 ± 38 | 5000 ± 93 | 1000 ± 40 | 2500 ± 130 |
| A559H/V610H | CO | 0.70 ± 0.07 | 2000 ± 23.1 | 2870 ± 33.1 | 0.082 ± 0.008 | 3100 ± 150 | 4400 ± 220 | 38000 ± 1200 | 55000 ± 5900 |
|  | EV |  | 2000 ± 45.8 | 2870 ± 65.6 | 1.7 ± 0.4 | 2200 ± 210 | 3200 ± 290 | 1300 ± 330 | 1900 ± 470 |
| A559H/V610W | CO | 0.50± 0.03 | 1500 ± 3.5 | 2980 ± 6.9 | 0.052 ± 0.012 | 1500 ± 150 | 3000 ± 290 | 29000 ± 720 | 57000 ± 14000 |
|  | EV |  | 1600 ± 17.3 | 3170 ± 34.3 | 2.1 ± 0.3 | 1500 ± 84 | 3000 ± 170 | 710 ± 11.0 | 1400 ± 220 |
| A559W/V610H | CO | 0.51 ± 0.01 | 2800 ± 81.9 | 5500 ± 161 | 0.091 ± 0.021 | 4700 ± 530 | 9200 ± 1000 | 52000 ± 1300 | 100000 ± 26000 |
|  | EV |  | 2800 ± 53.3 | 5500 ± 104.8 | 2.7 ± 0.5 | 3700 ± 300 | 7300 ± 590 | 1400 ± 280 | 2700 ± 540 |
| A559W/V610W | CO | 0.53 ± 0.01 | 2100 ± 110.1 | 3990 ± 209.4 | 0.058 ± 0.017 | 2600 ± 320 | 5000 ± 610 | 45000 ± 1400 | 85000 ± 27000 |
|  | EV |  | 2700 ± 28.9 | 5140 ± 55 | 1.8 ± 0.2 | 2900 ± 110 | 5500 ± 210 | 1600 ± 190 | 3100 ± 360 |

^a^ Specific activity was measured as CO oxidation (or EV reduction) under standard assay conditions at 30°C, pH 8.0.

^b^ Normalized specific activity is the specific activity divided by Ni content.

^c^ *K*_m_ was determined by nonlinear fitting of the Michaelis–Menten equation using five (CO) or seven (EV) substrate concentrations, calculated with SigmaPlot 10.

^d^ *k*_cat_ was calculated from *V*_max_ values based on the enzyme concentration determined after Ni-NTA purification.

^e^ *k*_cat_^n^ is the Ni-normalized turnover number, calculated by dividing *k*_cat_ by Ni content.

Most values are rounded to the nearest 100.

**Table S6 | Data collection and structure refinement of *Ch*CODH2 WT and variants.**

**a. Anaerobic *Ch*CODH2 WT and variants.**

|  | **Anaerobic**  **A559W/V610H**  **PDB : 9IYM** | | **Anaerobic**  **A559H/V610H**  **PDB : 9IYN** | | **Anaerobic**  **A559W/V610W**  **PDB : 9IYO** | | **Anaerobic**  **WT**  **PDB : 9IYL** |
| --- | --- | --- | --- | --- | --- | --- | --- |
| ***Data collection*** |  | |  | |  | |  |
| Space group | *C*2 | | *C*2 | | *C*2 | | *C*2 |
| Wavelength (Å) | 1.0000 | | 1.0000 | | 1.0000 | | 1.0000 |
| Cell dimensions |  | |  | |  | |  |
| *a*, *b*, *c* (Å) | 111.7, 75.8, 71.4 | | 111.9, 75.3, 70.9 | | 112.0, 75.5, 71.5 | | 111.9, 75.8, 71.7 |
| α, β, γ (°) | 90.0, 111.3, 90.0 | | 90.0, 111.2, 90.0 | | 90.0, 111.8, 90.0 | | 90.0, 111.6, 90.0 |
| Resolution (Å) | 50-1.82 (1.85-1.82)^a^ | | 50-1.83 (1.86-1.83)^a^ | | 50-2.0 (2.03-2.00)^a^ | | 50-1.28 (1.36-1.28)^a^ |
| Total / Unique reflections | 333,121 / 50,080 | | 315,858 / 47,694 | | 250,025 / 37,021 | | 640,330 / 137,193 |
| *R*_merge_ (%) | 8.2 (88.9)^a^ | | 8.8 (71.9)^a^ | | 8.3 (69.2)^a^ | | 5.5 (69.8)^a^ |
| *R*_pim_ (%) | 5.0 (45.1)^a^ | | 3.3 (14.3)^a^ | | 5.0 (37.2)^a^ | | 3.2 (35.3)^a^ |
| *CC*_1/2_ (%) | 99.8 (77.5)^a^ | | 99.5 (81.8)^a^ | | 99.2 (84.1)^a^ | | 99.9 (72.1)^a^ |
| *I* / σ*I* | 16.1 (1.3)^a^ | | 20.1 (1.7)^a^ | | 20.3 (2.4)^a^ | | 13.4 (1.7)^a^ |
| Completeness (%) | 100 (100)^a^ | | 98.7 (90.4)^a^ | | 99.9 (100)^a^ | | 96.3 (92.8)^a^ |
| Redundancy | 6.7 (6.7)^a^ | | 6.6 (5.3)^a^ | | 6.8 (6.7)^a^ | | 4.7 (4.6)^a^ |
| ***Model refinement statistics*** | |  | |  | |  |  |
| Resolution range (Å) | 37.87-1.81 (1.85-1.81)^a^ | | 35.22-1.83 (1.87-1.83)^a^ | | 33.2-2.00 (2.06-2.00)^a^ | | 37.9-1.28 (1.29-1.28)^a^ |
| *R*_work_ / *R*_free_ (%) | 16.7 / 20.8 | | 16.8 / 22.0 | | 16.7 / 22.0 | | 17.5 / 19.5 |
| No. atoms |  | |  | |  | |  |
| Protein | 4,671 | | 4,667 | | 4,692 | | 4,659 |
| Cluster | 22 | | 22 | | 22 | | 22 |
| Water | 313 | | 336 | | 254 | | 494 |
| Average *B*-factor (Å^2^) |  | |  | |  | |  |
| Protein | 27.0 | | 22.0 | | 28.3 | | 18.7 |
| Cluster | 28.3 | | 23.0 | | 25.0 | | 18.5 |
| Water | 32.4 | | 27.0 | | 31.1 | | 27.9 |
| Clash score | 9.3 | | 7.7 | | 9.3 | | 6.9 |
| RMSDs from ideal geometry | |  | |  | |  |  |
| Bond lengths (Å) | 0.010 | | 0.009 | | 0.008 | | 0.008 |
| Bond angles (°) | 1.142 | | 1.092 | | 0.984 | | 1.088 |
| Ramachandran |  | |  | |  | |  |
| Preferred (%) | 96.2 | | 96.7 | | 96.7 | | 96.8 |
| Allowed (%) | 3.5 | | 3.0 | | 3.0 | | 2.8 |
| Outliers (%) | 0.3 | | 0.3 | | 0.3 | | 0.3 |

^a^Values in parentheses refer to the highest resolution shell.

**b. Air-exposed *Ch*CODH2 WT and variants.**

|  | **Air exposure 1 h**  **A559W/V610H**  **PDB : 9IYR** | | **Air exposure 2 h**  **A559W/V610H**  **PDB : 9IYS** | | **Air exposure 8 h**  **A559W/V610H**  **PDB : 9IYT** | **Air exposure 24 h**  **A559W/V610H**  **PDB : 9IYU** | | **Air exposure 2h**  **WT**  **PDB : 9IYV** |
| --- | --- | --- | --- | --- | --- | --- | --- | --- |
| ***Data collection*** |  | |  | |  |  | |  |
| Space group | *C*2 | | *C*2 | | *C*2 | *C*2 | | *C*2 |
| Wavelength (Å) | 1.0000 | | 1.0000 | | 1.0000 | 1.0000 | | 1.0000 |
| Cell dimensions |  | |  | |  |  | |  |
| *a*, *b*, *c* (Å) | 112.0, 76.2, 71.6 | | 111.3, 76.5, 71.1 | | 112.0, 76.0, 71.4 | 112.6, 76.3, 72.1 | | 112.0, 76.0, 71.1 |
| α, β, γ (°) | 90.0, 111.6, 90.0 | | 90.0, 111.1, 90.0 | | 90.0, 111.7, 90.0 | 90.0, 112.4, 90.0 | | 90.0, 111.4, 90.0 |
| Resolution (Å) | 50-1.45 (1.48-1.45)^a^ | | 50-1.55 (1.58-1.55)^a^ | | 50-2.00 (2.03-2.00)^a^ | 50-2.00 (2.12-2.00)^a^ | | 50-2.50 (2.65-2.50)^a^ |
| Total / Unique reflections | 670,056 / 97,777 | | 518,883 / 78,964 | | 247,617 / 37,835 | 230,755 / 37,984 | | 116,983 / 19,232 |
| *R*_merge_ (%) | 5.8 (68.7)^a^ | | 6.7 (65.1)^a^ | | 8.9 (62.8)^a^ | 6.5 (41.5)^a^ | | 3.8 (33.4)^a^ |
| *R*_pim_ (%) | 3.2 (37.7)^a^ | | 3.8 (39.3)^a^ | | 3.4 (22.5)^a^ | 3.5 (16.2)^a^ | | 2.5 (20.1)^a^ |
| *CC*_1/2_ (%) | 99.6 (85.0)^a^ | | 100 (84.2)^a^ | | 99.4 (85.0)^a^ | 99.8 (93.5)^a^ | | 99.9 (96.5)^a^ |
| *I* / σ*I* | 36.5 (1.8)^a^ | | 29.7 (1.5)^a^ | | 29.1 (2.2)^a^ | 16.3 (4.0)^a^ | | 26.41 (5.36)^a^ |
| Completeness (%) | 98.7 (97.2)^a^ | | 98.1 (94.7)^a^ | | 99.9 (99.9)^a^ | 99.0 (98.5)^a^ | | 98.7 (98.1)^a^ |
| Redundancy | 6.9 (6.2)^a^ | | 6.6 (5.6)^a^ | | 6.5 (6.5)^a^ | 6.1 (6.2)^a^ | | 6.1 (6.4)^a^ |
| ***Model refinement statistics*** | |  | |  | | |  |  |
| Resolution range (Å) | 33.30-1.45 (1.47-1.45)^a^ | | 33.17-1.55 (1.57-1.55)^a^ | | 33.13-1.99 (2.04-1.99)^a^ | 35.08-2.00 (2.05-2.00)^a^ | | 34.15-2.50 (2.63-2.50)^a^ |
| *R*_work_ / *R*_free_ (%) | 18.0 / 20.6 | | 17.4 / 20.2 | | 16.6 / 21.0 | 18.6 / 22.2 | | 19.2 / 26.5 |
| No. atoms |  | |  | |  |  | |  |
| Protein | 4,674 | | 4,671 | | 4,674 | 4,671 | | 4,653 |
| Cluster | 22 | | 22 | | 22 | 22 | | 12 |
| Water | 358 | | 369 | | 223 | 179 | | 15 |
| Average *B*-factor (Å^2^) |  | |  | |  |  | |  |
| Protein | 24.0 | | 26.9 | | 40.1 | 37.6 | | 63.3 |
| Cluster | 21.4 | | 25.26 | | 40.6 | 37.5 | | 64.8 |
| Water | 30.4 | | 32.8 | | 41.0 | 36.8 | | 58.1 |
| Clash score | 8.1 | | 7.7 | | 7.9 | 6.8 | | 7.8 |
| RMSDs from ideal geometry | |  | |  | | |  |  |
| Bond lengths (Å) | 0.009 | | 0.008 | | 0.010 | 0.011 | | 0.008 |
| Bond angles (°) | 1.088 | | 1.081 | | 1.114 | 1.192 | | 0.944 |
| Ramachandran |  | |  | |  |  | |  |
| Preferred (%) | 97.2 | | 96.5 | | 96.8 | 96.4 | | 94.8 |
| Allowed (%) | 2.5 | | 3.2 | | 2.8 | 3.5 | | 4.8 |
| Outliers (%) | 0.3 | | 0.53 | | 0.3 | 0.2 | | 0.5 |

^a^Values in parentheses refer to the highest resolution shell.

| **Table S7 \| Anomalous data collection.** | | | | | |  |
| --- | --- | --- | --- | --- | --- | --- |
|  | **Anaerobic A559W/V610H**  **Fe anomalous** | **Anaerobic A559W/V610H**  **Ni anomalous** | **Anaerobic A559W/V610W**  **Fe anomalous** | **Anaerobic A559H/V610H**  **Fe anomalous** | **Anaerobic WT**  **Fe anomalous** | |
| ***Data collection*** |  |  |  |  |  | |
| Space group | *C*2 | *C*2 | *C*2 | *C*2 | *C*2 | |
| Cell dimensions |  |  |  |  |  | |
| *a*, *b*, *c* (Å) | 112.2, 76.2, 71.8 | 112.1, 76.1, 71.8 | 112.4, 76.2, 72.2 | 112.1, 75.6, 71.4 | 112.4, 75.4, 71.5 | |
| α, β, γ (°) | 90.0, 112.0, 90.0 | 90.0, 112.1, 90.0 | 90.0, 112.2, 90.0 | 90.0, 111.4, 90.0 | 90.0, 111.6, 90.0 | |
| Wavelength (Å) | 1.7364 | 1.4806 | 1.7364 | 1.7364 | 1.7364 | |
| Resolution (Å) | 50–2.85 (3.02-2.85)^a^ | 50-2.80 (2.97-2.80)^a^ | 50-2.89 (3.07-2.89)^a^ | 50-2.20 (2.33-2.20)^a^ | 50-1.99 (2.11-1.99)^a^ | |
| *R*_merge_ (%) | 4.0 (6.8)^a^ | 7.0 (19.9)^a^ | 4.3 (7.5)^a^ | 5.1 (25.5)^a^ | 3.9 (25.1)^a^ | |
| *R*_pim_ (%) | 3.6 (5.3)^a^ | 3.2 (14.9)^a^ | 3.6 (6.4)^a^ | 2.9 (16.9)^a^ | 2.7 (10.4)^a^ | |
| *CC*_1/2_ (%) | 99.6 (99.0) ^a^ | 99.2 (92.6)^a^ | 99.6 (99.0)^a^ | 99.7 (94.8)^a^ | 99.9 (94.5)^a^ | |
| *I* / σ*I* | 12.2 (18.7)^a^ | 10.2 (4.4)^a^ | 17.0 (10.0)^a^ | 15.0 (4.0)^a^ | 20.1 (4.3)^a^ | |
| Completeness (%) | 94.6 (94.7)^a^ | 96.4 (91.5)^a^ | 92.6 (93.0)^a^ | 98.9 (96.0)^a^ | 97.5 (94.6)^a^ | |
| Redundancy | 2.3 (2.3)^a^ | 2.34 (2.2)^a^ | 2.4 (2.4)^a^ | 3.5 (3.4)^a^ | 3.4 (3.4)^a^ | |
|  | **Anaerobic WT**  **Ni anomalous** | **Air exposure 1 h A559W/V610H**  **Fe anomalous** | **Air exposure 1h A559W/V610H**  **Ni anomalous** | **Air exposure 2 h A559W/V610H**  **Fe anomalous** | **Air exposure 2 h A559W/V610H**  **Ni anomalous** | |
| ***Data collection*** |  |  |  |  |  | |
| Space group | *C*2 | *C*2 | *C*2 | *C*2 | *C*2 | |
| Cell dimensions |  |  |  |  |  | |
| *a*, *b*, *c* (Å) | 112.3, 75.4, 71.6 | 112.0, 75.7, 71.6 | 112.0, 75.8, 71.4 | 112.1, 76.0, 71.7 | 111.9, 75.7, 71.3 | |
| α, β, γ (°) | 90.0, 111.5, 90.0 | 90.0, 111.5, 90.0 | 90.0, 111.5, 90.0 | 90.0, 111.8, 90.0 | 90.0, 111.5, 90.0 | |
| Wavelength (Å) | 1.4852 | 1.7364 | 1.4852 | 1.7364 | 1.4852 | |
| Resolution (Å) | 50–2.09 (2.22-2.09)^a^ | 50-2.39 (2.54-2.39)^a^ | 50-1.80 (1.90-1.80)^a^ | 50-1.79 (1.90-1.79)^a^ | 50-1.89 (2.01-1.89)^a^ | |
| *R*_merge_ (%) | 4.2 (10.4)^a^ | 9.2 (19.5)^a^ | 4.4 (19.8)^a^ | 4.8 (26.9)^a^ | 3.7 (20.6)^a^ | |
| *R*_pim_ (%) | 2.3 (7.9)^a^ | 5.4 (10.5)^a^ | 2.9 (14.8)^a^ | 2.7 (9.7)^a^ | 2.2 (8.9)^a^ | |
| *CC*_1/2_ (%) | 99.8 (98.6)^a^ | 98.8 (94.5)^a^ | 99.8 (95.5)^a^ | 99.8 (92.5)^a^ | 99.9 (96.4)^a^ | |
| *I* / σ*I* | 20.0 (8.2)^a^ | 22.0 (12.7)^a^ | 17.4 (4.4)^a^ | 14.4 (3.16)^a^ | 20.0 (5.0)^a^ | |
| Completeness (%) | 98.1 (98.8)^a^ | 98.5 (95.7)^a^ | 96.6 (94.5)^a^ | 98.3 (94.6)^a^ | 99.3 (99.0)^a^ | |
| Redundancy | 3.4 (3.4)^a^ | 3.4 (3.3)^a^ | 3.3 (2.8)^a^ | 3.3 (3.1)^a^ | 3.4 (3.4)^a^ | |
|  | **Air exposure 8 h A559W/V610H**  **Fe anomalous** | **Air exposure 8 h A559W/V610H**  **Ni anomalous** | **Air exposure 24 h A559W/V610H**  **Fe anomalous** | **Air exposure 24 h A559W/V610H**  **Ni anomalous** | **Air exposure 2 h WT**  **Fe anomalous** | |
| ***Data collection*** |  |  |  |  |  | |
| Space group | *C*2 | *C*2 | *C*2 | *C*2 | *C*2 | |
| Cell dimensions |  |  |  |  |  | |
| *a*, *b*, *c* (Å) | 112.4, 76.3, 72.0 | 112.0, 76.0, 71.6 | 112.2, 76.0, 71.7 | 112.3, 76.3, 71.9 | 111.6, 76.9, 70.8 | |
| α, β, γ (°) | 90.0, 111.1, 90.0 | 90.0, 111.6, 90.0 | 90.0, 111.8, 90.0 | 90.0, 111.8, 90.0 | 90.0, 111.3, 90.0 | |
| Wavelength (Å) | 1.7364 | 1.4852 | 1.7364 | 1.4852 | 1.7364 | |
| Resolution (Å) | 50-2.20 (2.33-2.20)^a^ | 50-1.90 (2.01-1.90)^a^ | 50-2.30 (2.44-2.30)^a^ | 50-2.30 (2.44-2.30)^a^ | 50-2.59 (2.75-2.59)^a^ | |
| *R*_merge_ (%) | 5.6 (28.1)^a^ | 4.3 (13.0)^a^ | 3.2 (5.1)^a^ | 3.4 (12.9)^a^ | 6.2 (70.9)^a^ | |
| *R*_pim_ (%) | 3.1 (9.7)^a^ | 2.3 (6.3)^a^ | 2.8 (4.4)^a^ | 2.2 (6.1)^a^ | 3.4 (37.9)^a^ | |
| *CC*_1/2_ (%) | 99.7 (92.7)^a^ | 99.8 (98.2)^a^ | 99.8 (99.4)^a^ | 99.9 (98.5)^a^ | 99.9 (75.9)^a^ | |
| *I* / σ*I* | 13.9 (4.0)^a^ | 18.8 (7.1)^a^ | 21.7 (13.4)^a^ | 24.0 (8.0)^a^ | 13.7 (1.8)^a^ | |
| Completeness (%) | 98.9 (96.9)^a^ | 99.0 (98.1)^a^ | 91.3 (91.1)^a^ | 99.0 (98.4)^a^ | 99.4 (98.6)^a^ | |
| Redundancy | 3.4 (3.4)^a^ | 3.5 (3.5)^a^ | 2.4 (2.4)^a^ | 3.4 (3.4)^a^ | 3.5 (3.5)^a^ | |

^a^Values in parentheses refer to the highest resolution shell.

**Table S8 | S anomalous data collection.**

|  | **Anaerobic A559W/V610H**  **S anomalous** | **Air-exposed 2h A559W/V610H**  **S anomalous** |  |  |
| --- | --- | --- | --- | --- |
| ***Data collection*** |  |  |  |  |
| Space group | *C*2 | *C*2 |  |  |
| Cell dimensions |  |  |  |  |
| *a*, *b*, *c* (Å) | 111.8, 75.7, 71.5 | 112.1, 75.0, 71.6 |  |  |
| α, β, γ (°) | 90.0, 111.5, 90.0 | 90.0, 111.4, 90.0 |  |  |
| Wavelength (Å) | 1.8831 | 1.8831 |  |  |
| Resolution (Å) | 50-2.40 (2.54-2.40)^a^ | 50-2.30 (2.44-2.30)^a^ |  |  |
| *R*_merge_ (%) | 2.9 (6.6)^a^ | 2.1 (3.5)^a^ |  |  |
| *R*_pim_ (%) | 2.0 (3.8)^a^ | 1.5 (2.4)^a^ |  |  |
| *CC*_1/2_ (%) | 99.8 (99.1)^a^ | 99.9 (99.7)^a^ |  |  |
| *I* / σ*I* | 22.8 (10.34)^a^ | 32.15 (20.24)^a^ |  |  |
| Completeness (%) | 93.1 (90.3)^a^ | 91.2 (89.4)^a^ |  |  |
| Redundancy | 2.2 (2.0)^a^ | 2.2 (2.2)^a^ |  |  |
| ^a^Values in parentheses refer to the highest resolution shell. | | | | |


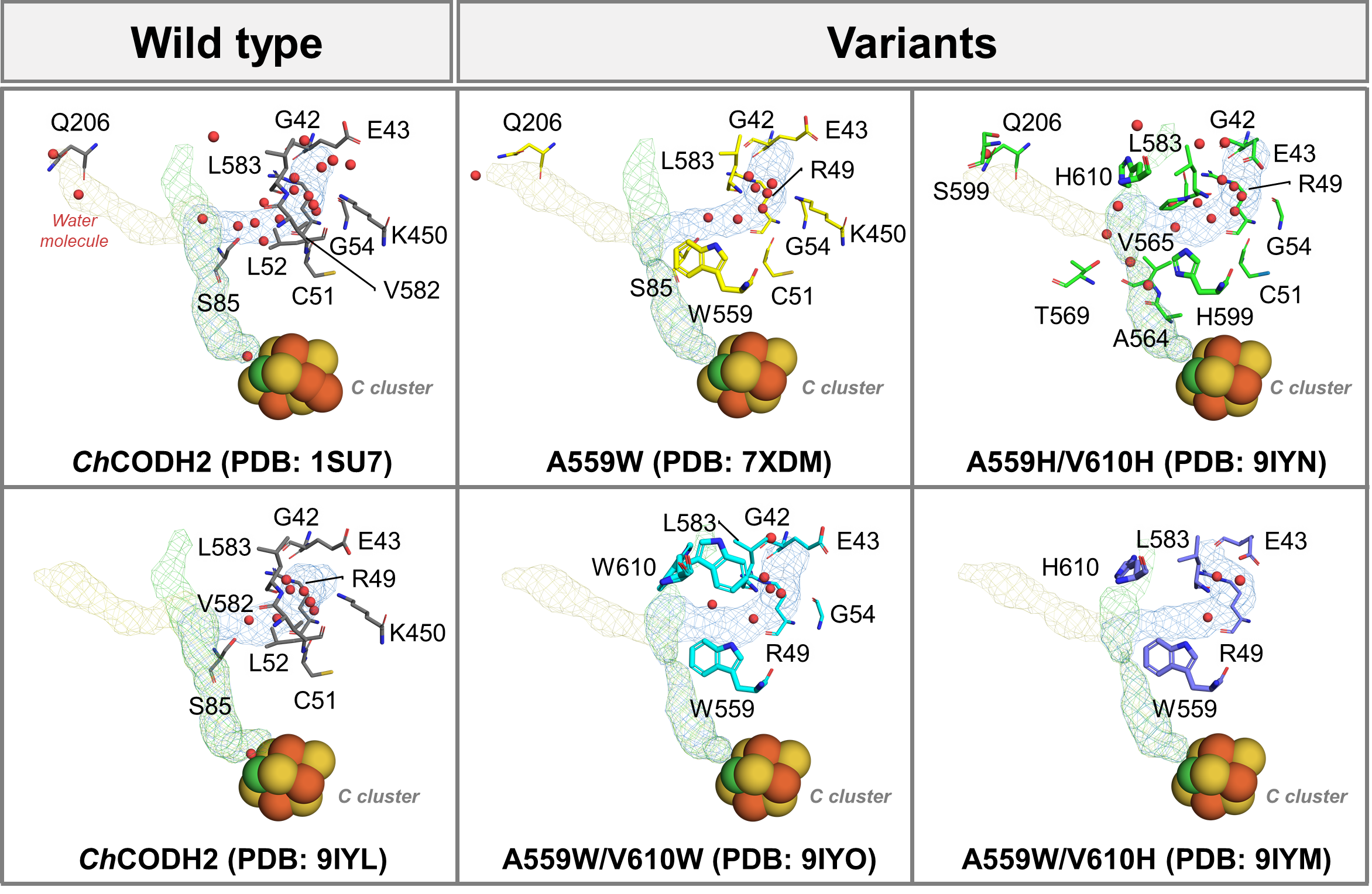


**Figure S1 | Water molecules and coordinating residues in the non-selective tunnels of *Ch*CODH2 WT and variants.** Water molecule positions (red spheres) and their interacting residues are shown along the non-selective tunnels in the wild-type and engineered *Ch*CODH2 variants. The left column shows wild-type *Ch*CODH2, and the two right columns represent engineered variants. Tunnel surfaces are color-coded: blue for tunnel #1, green for tunnel #2, and yellow for tunnel #3. Residues involved in water coordination are labeled. The comparison reveals that in the A559W/V610H variant (bottom right), water molecules are absent at the entrance of tunnel #2, suggesting that the narrowed tunnel restricts the passage of both water and oxygen molecules, which have similar van der Waals radii. These observations suggest that water molecules may serve as proxies for potential oxygen migration routes within the tunnel, as both share comparable molecular dimensions, despite differences in polarity. However, water distribution may vary depending on crystallization conditions and should therefore be considered a qualitative indicator rather than a definitive map of gas migration.


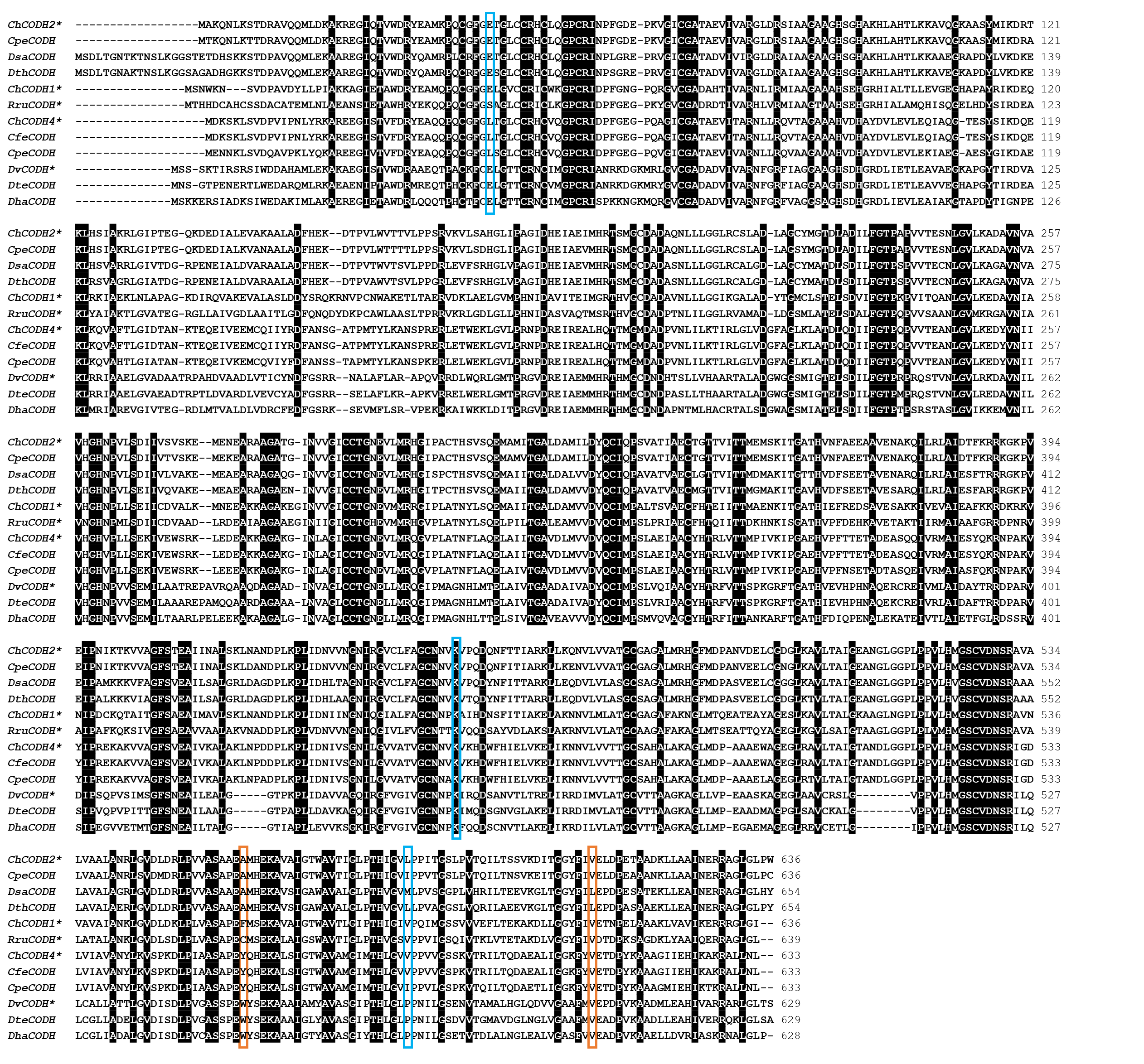


**Figure S2 | Sequence alignment of *Ch*CODH-related proteins.** Amino acid sequences of *Ch*CODH homologs were aligned to evaluate conservation at the mutation sites. Residues that are fully conserved (100% identity) are shown in black with white lettering. Functionally characterized CODHs are marked with asterisks (*). Orange boxes indicate key substituted residues A559 and V610, while blue boxes highlight additional mutated residues E43, K450, and L583 introduced in this study. These alignment results provide context for the functional and evolutionary relevance of the mutated positions. The aligned sequences include *Ch*CODH1 (WP_011344718), *Ch*CODH2 (WP_011343033), and *Ch*CODH4 (WP_011343666) from *Carboxydothermus hydrogenoformans*; *Dsa*CODH from *Desulfofundulus salinum* (WP_121452276); *Rru*CODH from *Rhodospirillum rubrum* (WP_011389181); *Dv*CODH from *Desulfovibrio vulgaris* (WP_010939375); *Dte*CODH from *Desulfofundulus termitidis* (WP_035067836); and *Dha*CODH from *Desulfofaba hansenii* (WP_100393885).


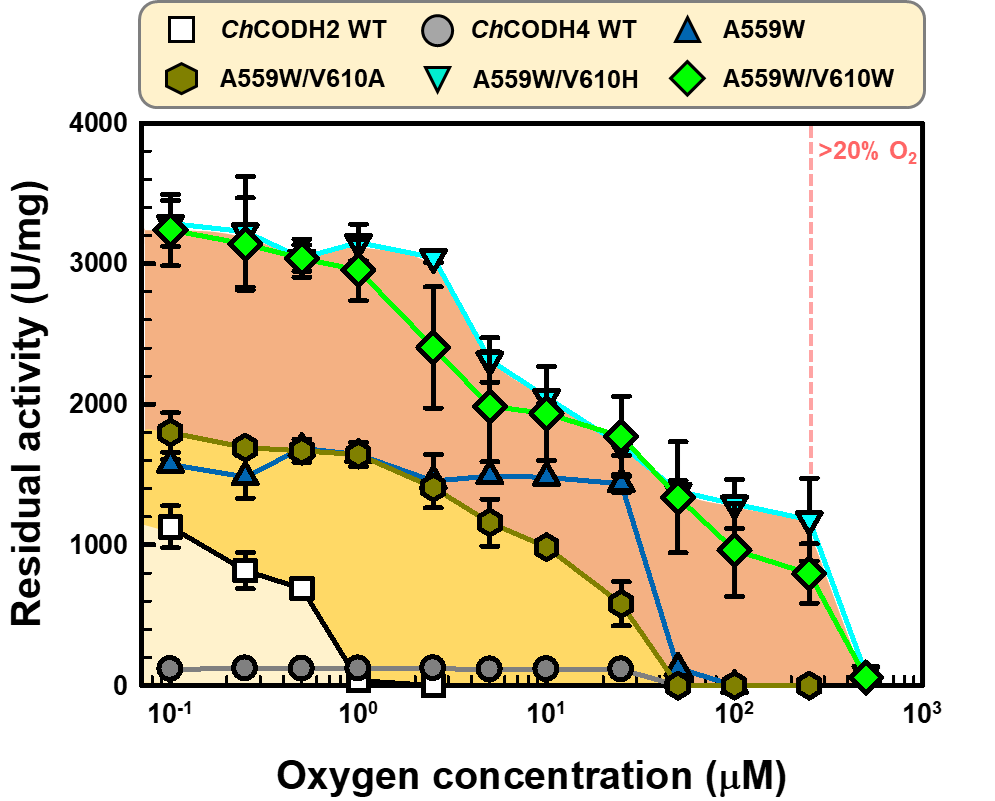


**Figure S3 | Specific activity of *Ch*CODH WT and variants at varying oxygen concentrations.** The changes in specific activity of *Ch*CODH WT and its variants at different oxygen concentrations are depicted. A general trend of decreased activity is observed for all CODH enzymes as oxygen concentration increases. A559W/V610H and A559W/V610W maintain higher residual activity, demonstrating enhanced oxygen resistance. Data represent the mean ± S.D., determined from *n* = 3 independent experiments. Specific activity (1 U/mg) is defined as 1 μmol reduced viologen·min^‒1^·mg^‒1^.

**
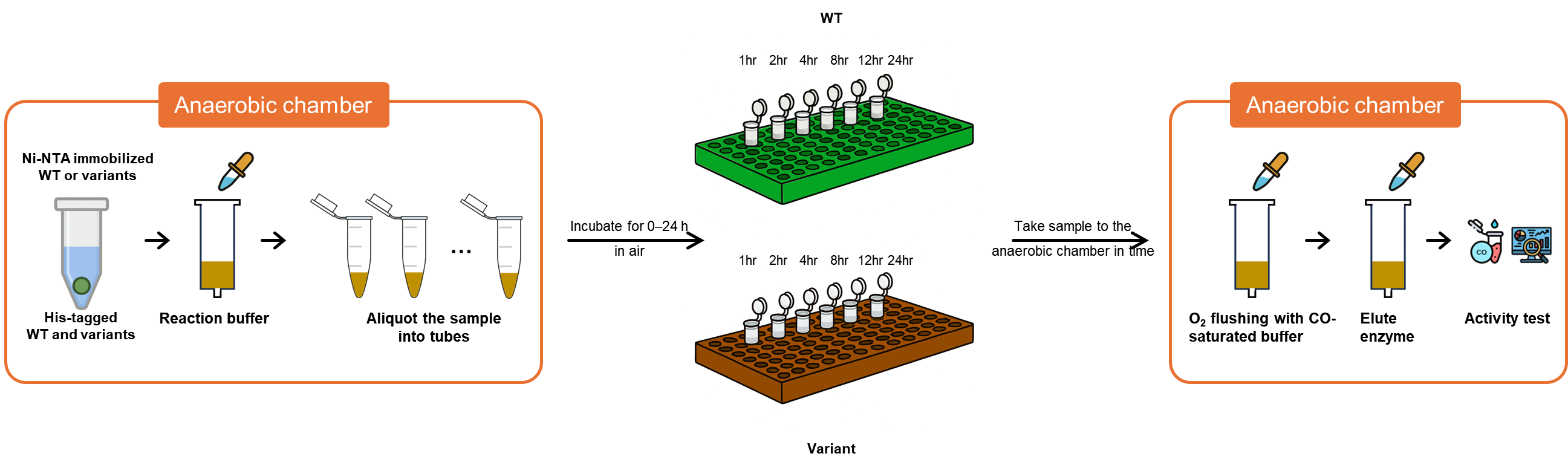
**

**Figure S4 | Workflow for evaluating air stability of Ni-NTA immobilized *Ch*CODH2 variants.** This schematic illustrates the experimental procedure used to assess the oxygen tolerance of *Ch*CODH2 wild-type (WT) and variant enzymes following air exposure. The workflow includes anaerobic immobilization, time-controlled aerobic exposure, oxygen removal, elution, and CO oxidation activity measurement. **Step 1. Anaerobic immobilization and sample prep**: His-tagged WT and variant enzymes were immobilized on Ni-NTA resin in an anaerobic chamber and aliquoted into sealed tubes for timed air exposure. **Step 2. Air exposure**: Samples were incubated in ambient air at room temperature for 0–24 h (five timepoints for WT and four double mutants). **Step 3. Oxygen removal and elution**: After exposure, samples were returned to the anaerobic chamber, washed with CO-saturated buffer to remove O_2_, and eluted to recover the active enzyme. **Step 4. Activity and protein assays**: Eluted enzymes were tested for CO oxidation activity and protein concentration under anaerobic conditions.

**Note:** Resazurin indicator was used in washing buffers to confirm complete oxygen removal by visual inspection (colorless = anaerobic).


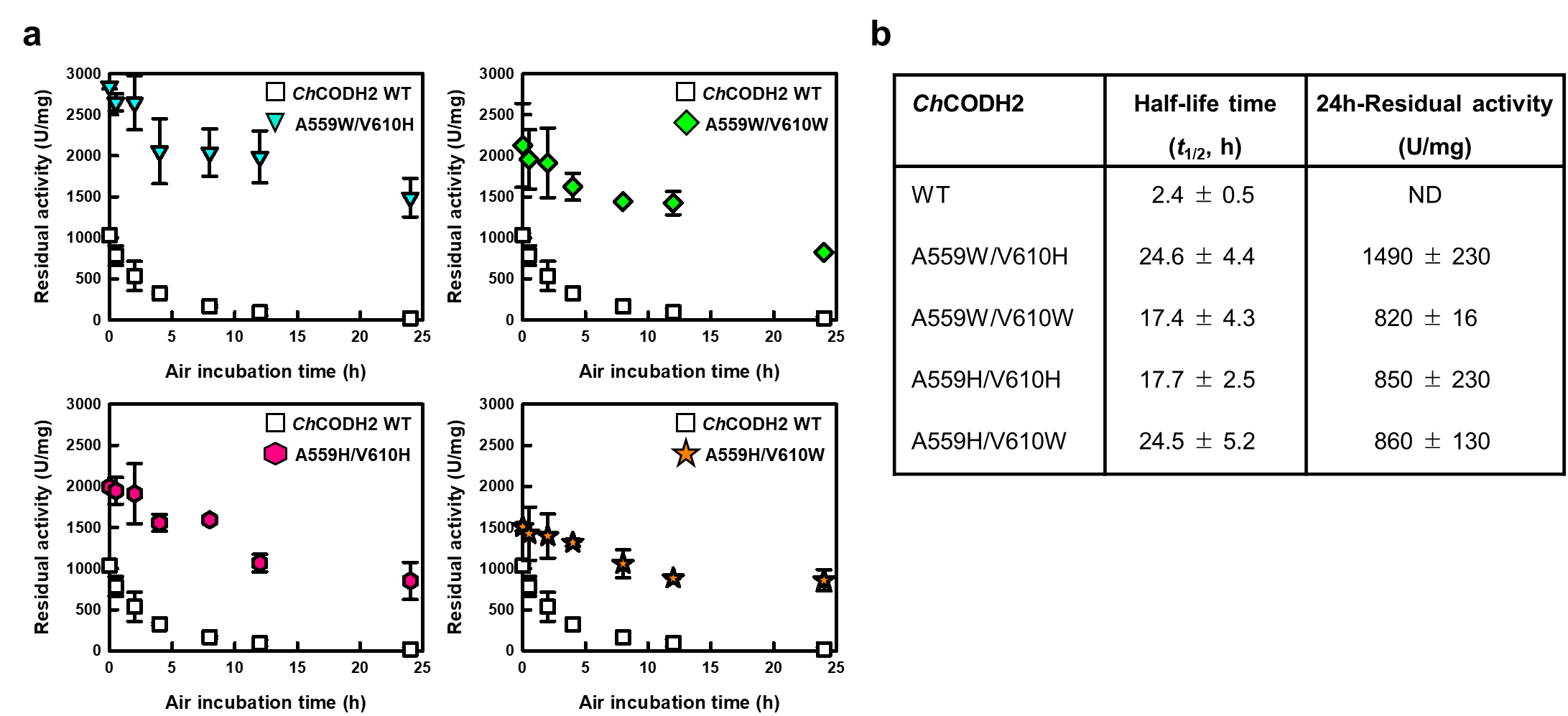


**Figure S5 | Residual activity and half-life time (*t*_1/2_) of *Ch*CODHs under atmospheric conditions.** (a) The residual activities. Specific activity (1 U/mg) is defined as 1 μmol reduced viologen·min^‒1^·mg^‒1^. (b) Half-life time (*t*_1/2_, hours). *Ch*CODH2 (squares), A559H/V610H (hexagons), A559H/V610W (stars), A559W/V610H (reverse triangles), and A559W/V610W (diamonds) in air were monitored over 24 hours. CODHs were exposed to air for a period of time and then re-purified under anaerobic conditions using immobilization on Ni-NTA resin. Data represent the mean ± S.D., determined from *n* = 3 independent experiments. 24h-residual activity means residual activity after 24 hours of air exposure; ND means not detected.

**
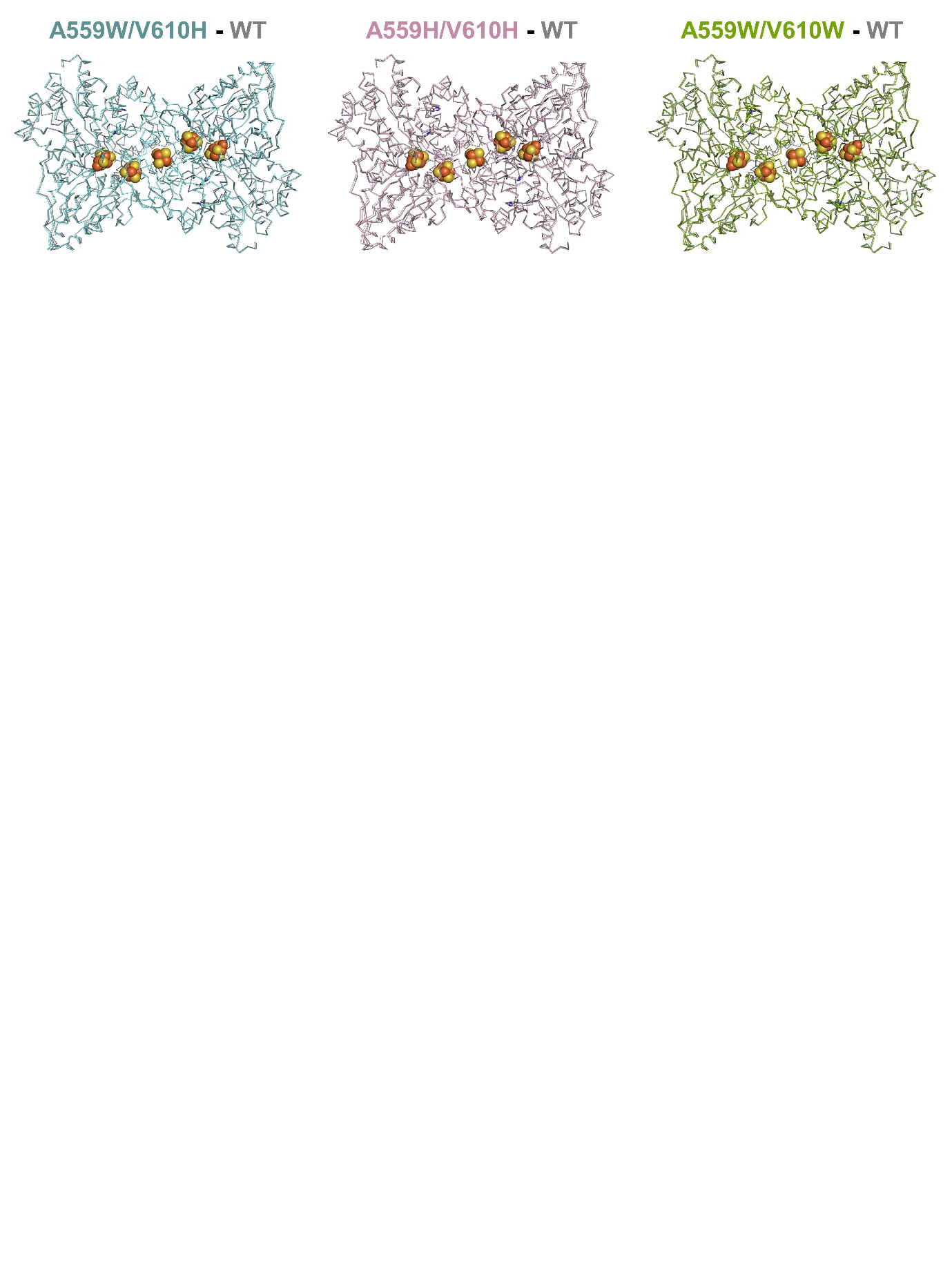
**

**Figure S6 | Structural differences of *Ch*CODH2 WT and variants.** Structural models of *Ch*CODH2 variants A559W/V610H, A559H/V610H, and A559W/V610W are depicted using a wireframe representation, which emphasizes the configuration of amino acids around the metal clusters. These clusters are represented as spheres, illustrating their spatial relationships within the enzyme's active site. Based on low values of root mean square deviations (RMSD) between the wild-type structure (PDB 1SU7) and each variant (0.319 Å in A559W/V610H, 0.323 Å in A559H/V610H and 0.308 Å in A559W/V610W for 633 superposed Cα atoms), it can be concluded that introduced substitutions did not disturb the overall structure.

**
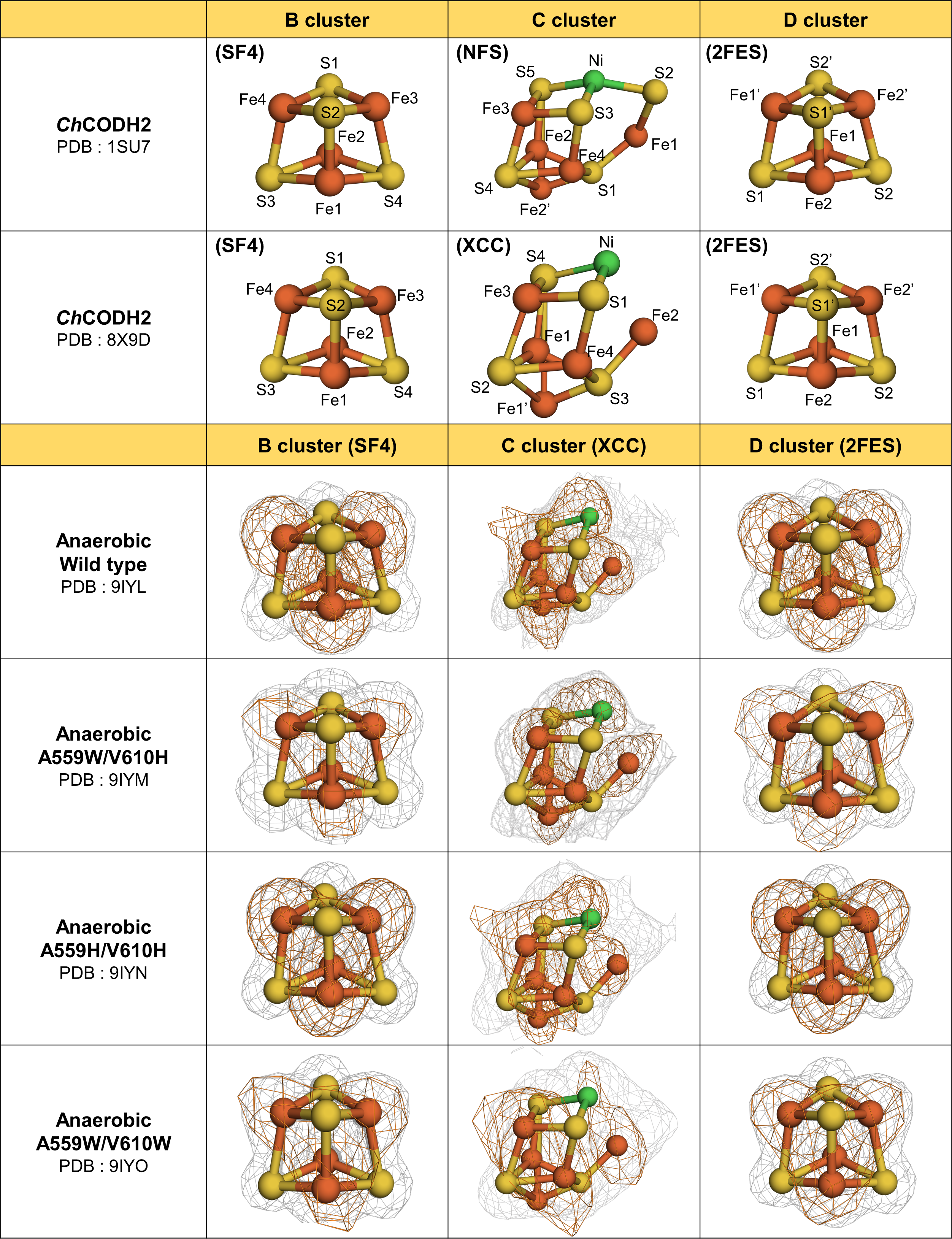
**

**Figure S7 | The *F_o_-F_c_* omit map and Fe anomalous maps for the B, C, and D-clusters in *Ch*CODH2 WT and variants.** The *F_o_-F_c_* omit maps of the B, C, and D clusters in the WT and double variants (A559W/V610H, A559H/V610H, A559W/V610W) under anaerobic conditions are contoured at 3.0σ (grey mesh), in which non-protein atoms were omitted in the calculation of *F_c_*. Fe, S, and Ni atoms are coloured in orange, yellow and green, respectively. For the C cluster, 1SU7^[10]^ and 8X9D^[11]^ exhibit the Ni-4Fe-5S (NFS) and Ni-4Fe-4S (XCC) conformations, respectively. The C-cluster of the double variants exhibit the Ni-4Fe-4S (XCC) conformation. The anomalous difference Fourier maps illustrating the positions of Fe atoms in B, C, and D clusters are shown as orange mesh, contoured at 8.0σ, 4.0σ, and 8.0σ for each cluster, respectively.

**
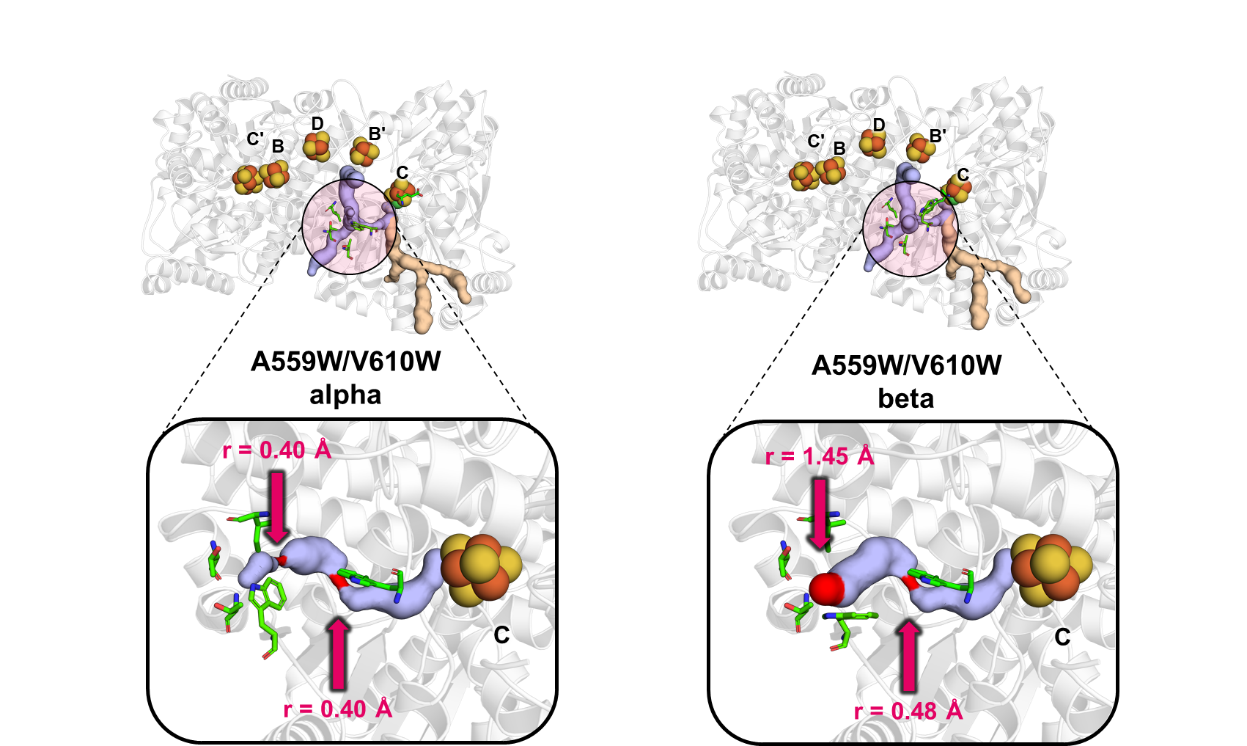
**

**Figure S8 | Gas tunnels of *Ch*CODH2 A559W/V610W.** Two alternative conformations of the V610W create gas tunnels, with the radius (*r*) at their bottlenecks indicated. The gas tunnels are shown as non-selective tunnels (light blue) and selective tunnels (light orange). The internal tunnels and bottleneck points (highlighted in red) of A559W/V610W are displayed, with the expanded *r* shown in black boxes.

**
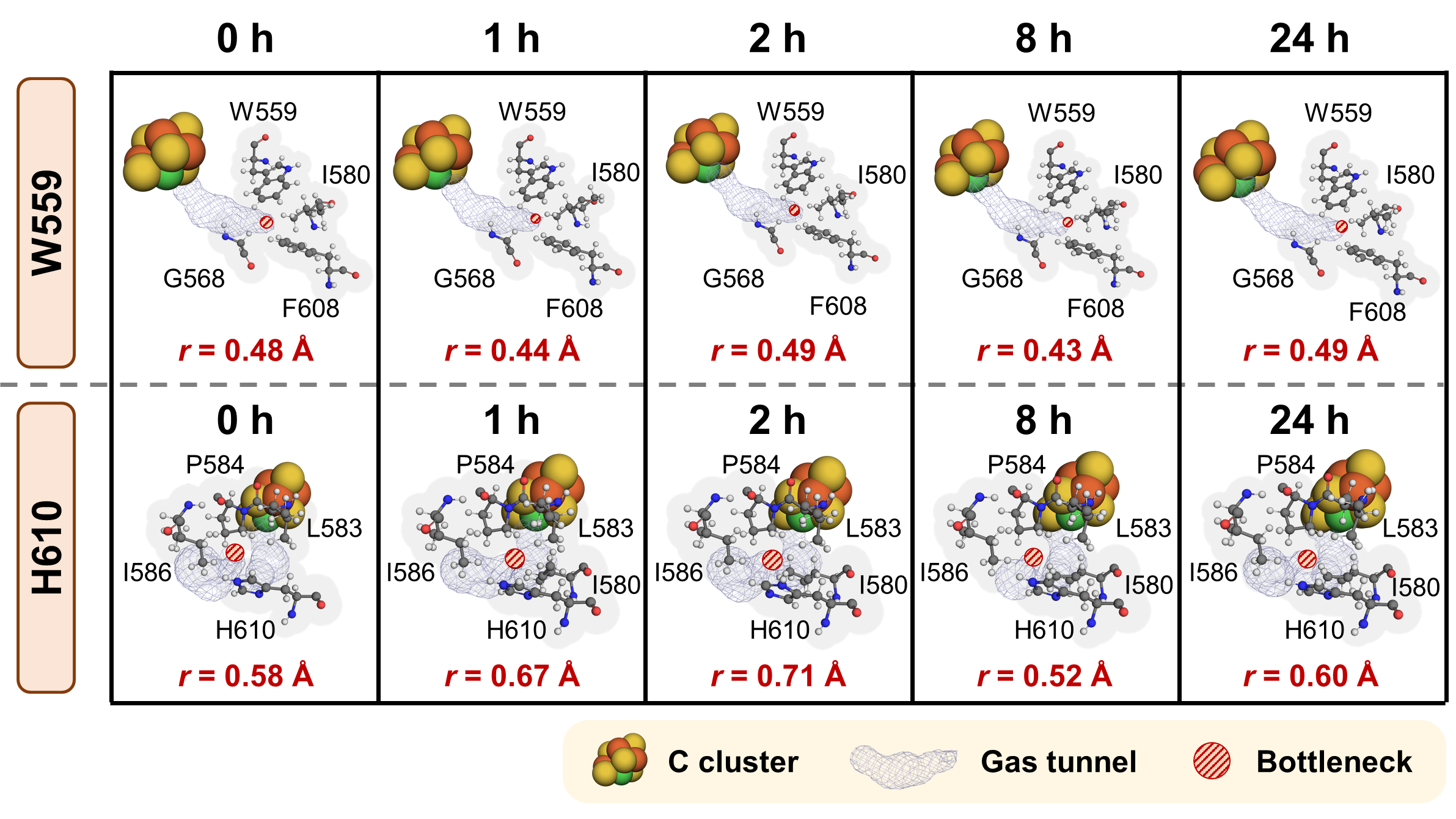
**

**Figure S9 | Changes in the tunnel radius of air-exposed *Ch*CODH2 A559W/V610H.** Bottleneck residues W559 and H610, along with the corresponding tunnel cross-sectional radius, are shown. The C cluster (spheres), gas tunnel (light blue mesh), and bottleneck (red hatch) are highlighted. Each time point illustrates the tunnel residues (ball and stick) and their van der Waals surfaces (grey shaded).

| **Cluster**  **(Conformation)** | **Air-exposed time** | | | | | | |
| --- | --- | --- | --- | --- | --- | --- | --- |
|  | **A559W/V610H** | | | | | **WT** | |
|  | **0 h**  **(9IYM)** | **1 h**  **(9IYR)** | **2 h**  **(9IYS)** | **8 h**  **(9IYT)** | **24 h**  **(9IYU)** | **0 h**  **(9IYL)** | **2 h**  **(9IYV)** |
| **B cluster**  **(SF4)** | **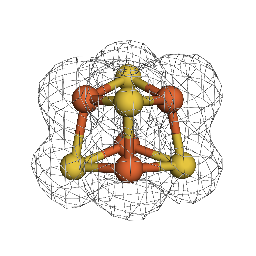** | **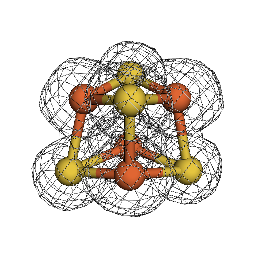** | **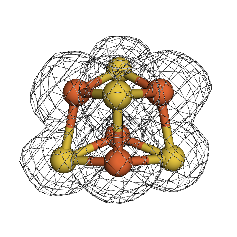** | **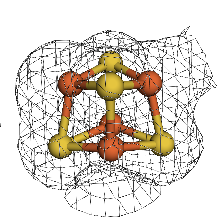** | **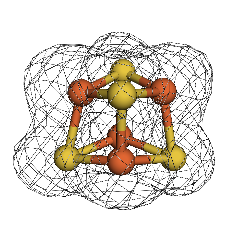** | 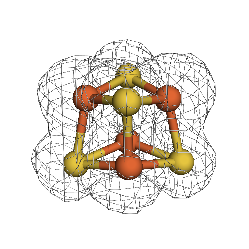 | **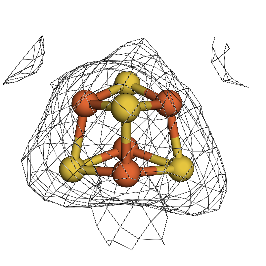** |
| **C cluster**  **(XCC)** | 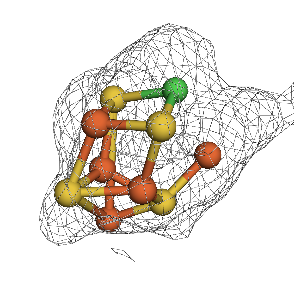 | 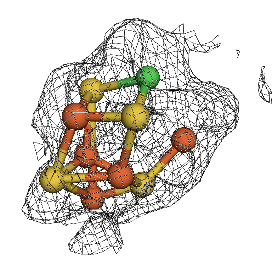 | 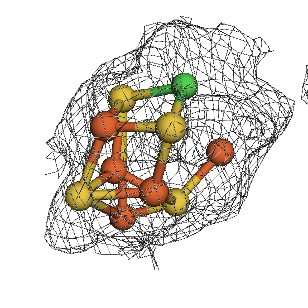 | 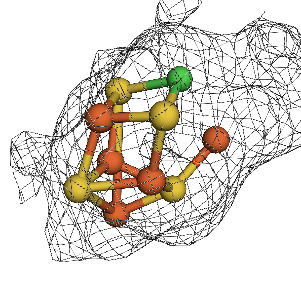 | 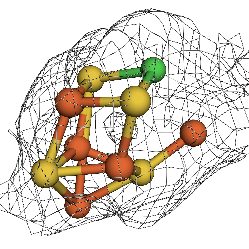 | 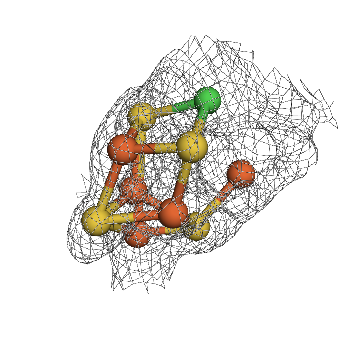 | **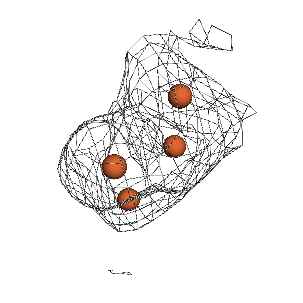** |
| **D cluster**  **(2FES)** | **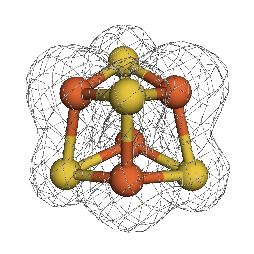** | **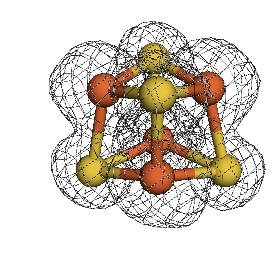** | **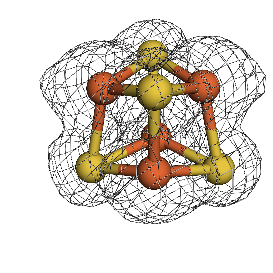** | **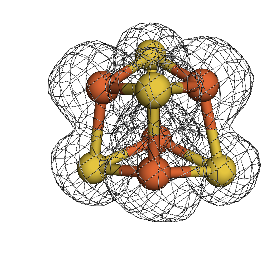** | **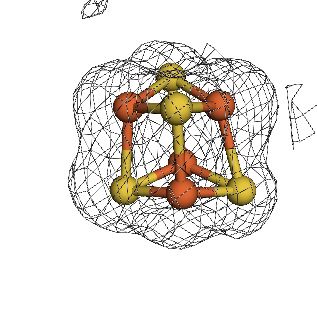** | **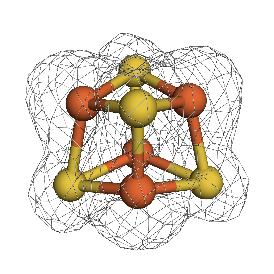** | **No map** |

**Figure S10 | Omit maps (*F_o_-F_c_*) for B, C, and D-clusters in *Ch*CODH2 WT and A559W/V610H variant over air exposure time.** Omit maps (*F_o_-F_c_*) of each cluster in the A559/V610H variant and WT after time-dependent oxygen exposure, contoured at 3.0σ and shown as a grey mesh, in which non-protein atoms were omitted in the calculation of *F_c_*. Fe, S, and Ni atoms are colored orange, yellow, and green, respectively. The C-cluster of both the A559W/V610H variant and WT adopts the Ni-4Fe-4S (XCC) conformation.

**
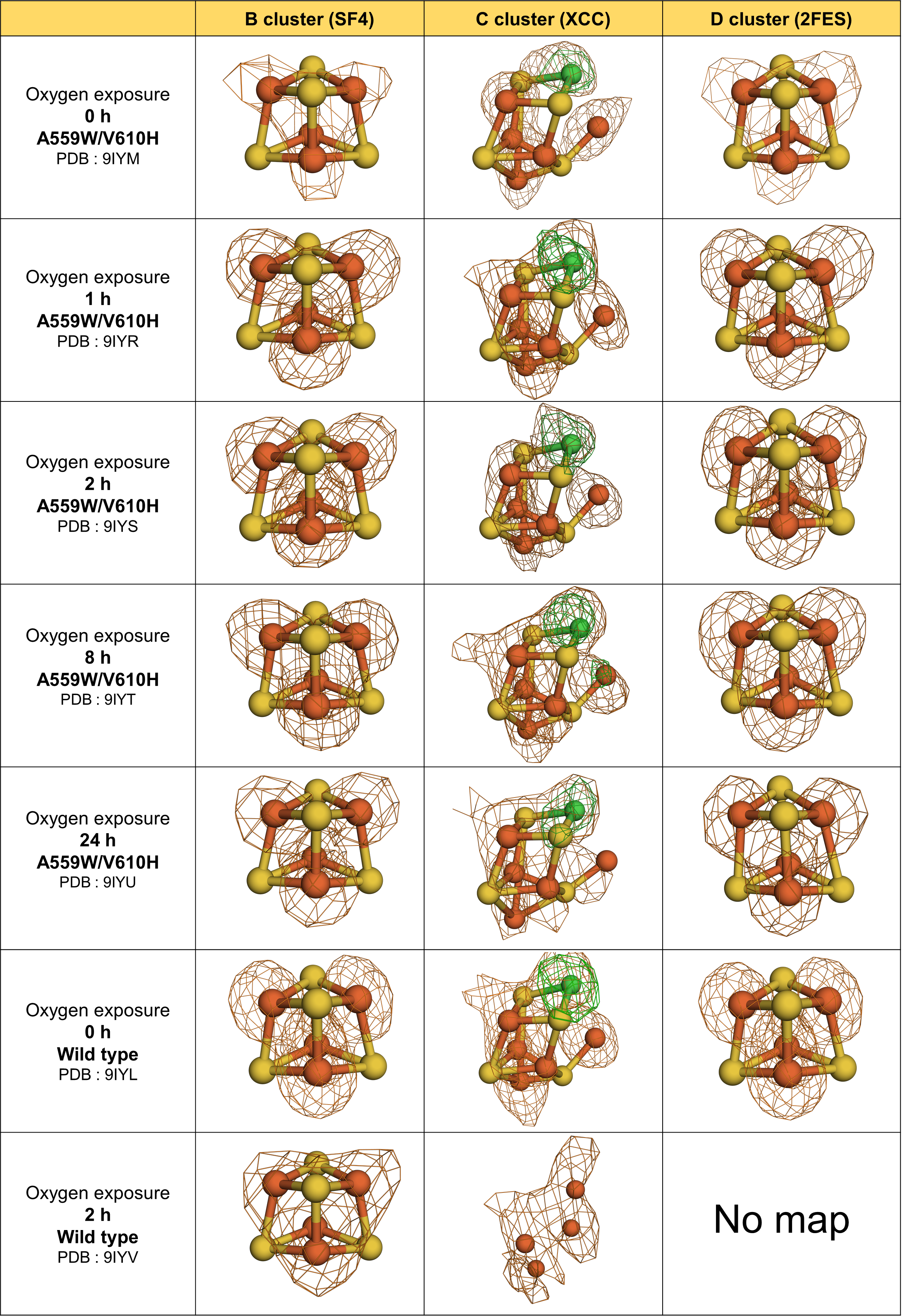
**

**Figure S11 | Fe and Ni anomalous difference Fourier maps after time-dependent air exposure.** Anomalous difference Fourier maps for Fe atoms in B, C, and D clusters are contoured at 9.0σ, 4.0σ and 8.0σ in each cluster, respectively, shown as orange mesh. Fe, S, and Ni atoms are coloured orange, yellow and green, respectively. Anomalous difference Fourier maps for Ni atoms in the C cluster are contoured at 3.8σ showed with green mesh.

**
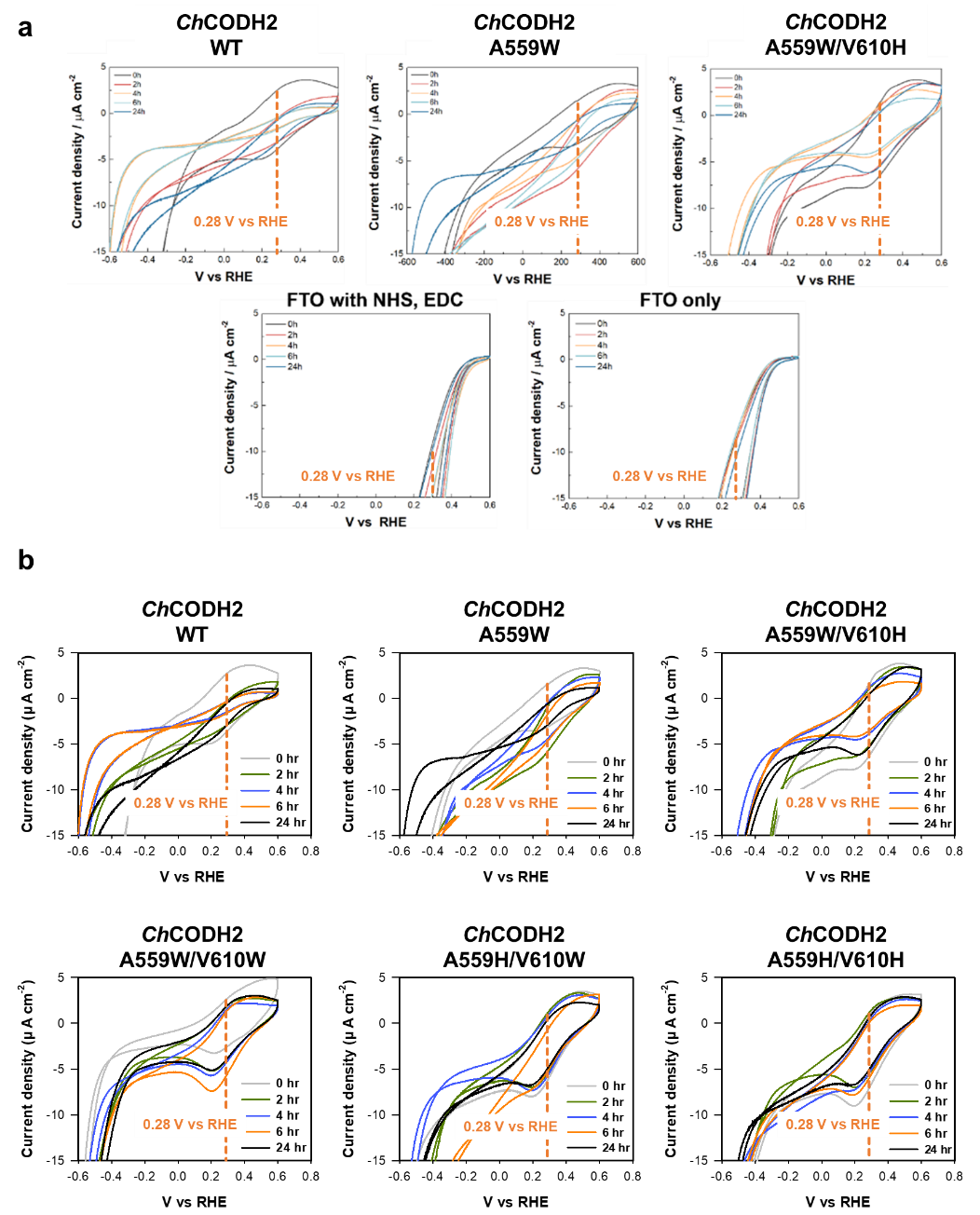
**

**Figure S12 | Electrode validation and electrochemical air stability of *Ch*CODH2 variants.** (a) Control experiments for electrode binding affinity. Control experiments were performed to verify that the observed differences in cyclic voltammetry (CV) responses among the enzyme variants were not due to variations in electrode binding affinity. CVs were recorded over time (0, 2, 4, 6, and 24 h) for wild-type (WT), A559W, and A559W/V610H variants immobilized on fluorine-tin oxide (FTO) electrodes, and compared with control electrodes treated with NHS/EDC chemistry (without protein) as well as bare FTO electrodes. All measurements were conducted under aerobic conditions. The current response at 0.28 V vs RHE (orange dashed line) remained negligible in the absence of enzyme, confirming that the catalytic signals originated from the protein variants themselves rather than from differential adsorption to the electrode surface. (b) Electrochemical CO oxidation of *Ch*CODH2 variants for 24 h air exposure. CVs were performed on CODH-immobilized FTO electrodes under 100% (*v*/*v*) CO, 25°C, and pH 8. Air exposure times matched those in panel (a): 0 h (grey), 2 h (green), 4 h (blue), 6 h (orange), and 24 h (black). The observed potential (V vs reversible hydrogen electrode (RHE)) in CO oxidation reactions is indicated by an orange dotted line at 0.28 V vs RHE. All measurements were conducted in triplicate to verify reproducible CV profiles.

**
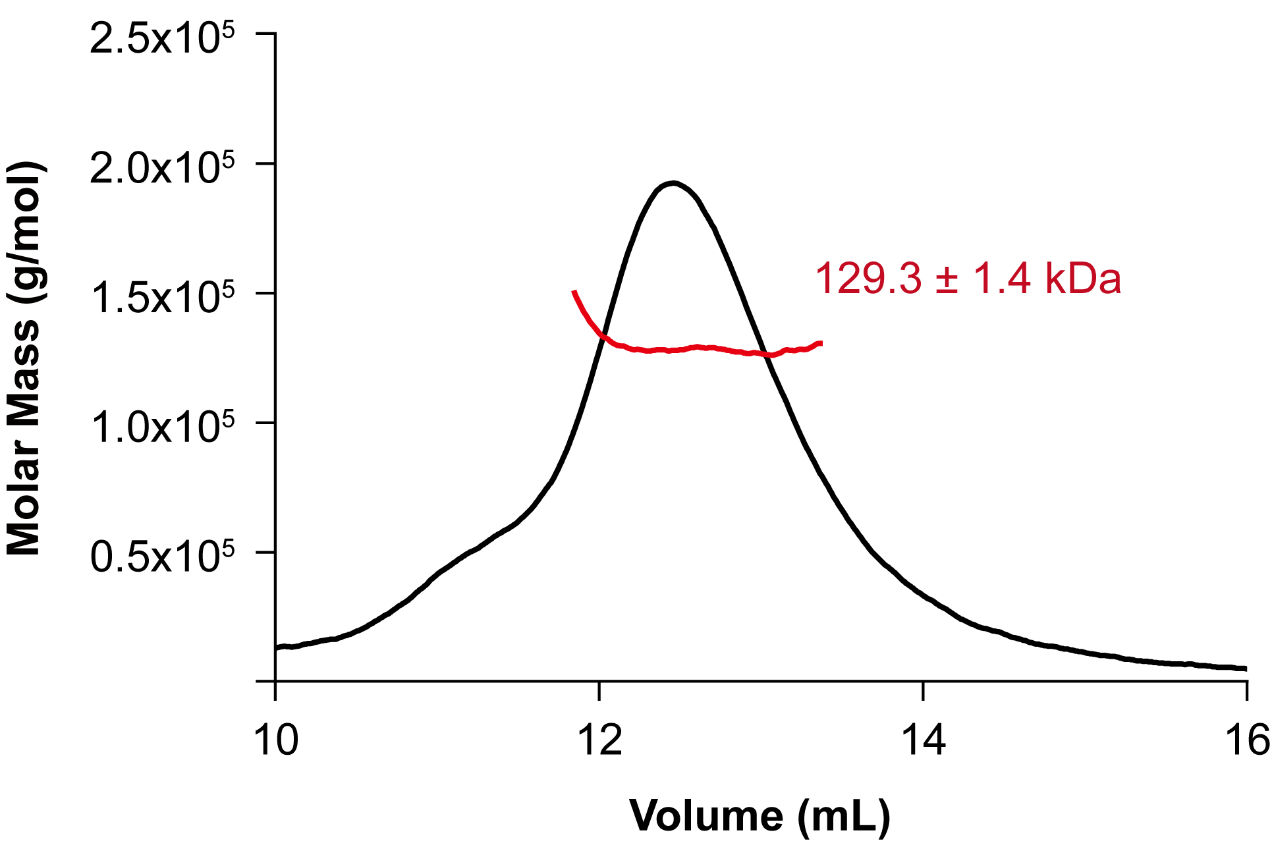
**

**Figure S13 | SEC-MALS analysis of *Ch*CODH2 A559W/V610H oligomeric state.** The dimeric state of the A559W/V610H variant (PDB ID: 9IYM) was analysed using size-exclusion chromatography with multi-angle light scattering (SEC-MALS) in solution. The horizontal line (red) represents the measured molecular mass (129.3 ± 1.4 kDa). Under air exposure conditions, the expected molecular mass for the dimeric *Ch*CODH2 A559W/V610H variant was 138 kDa.

**
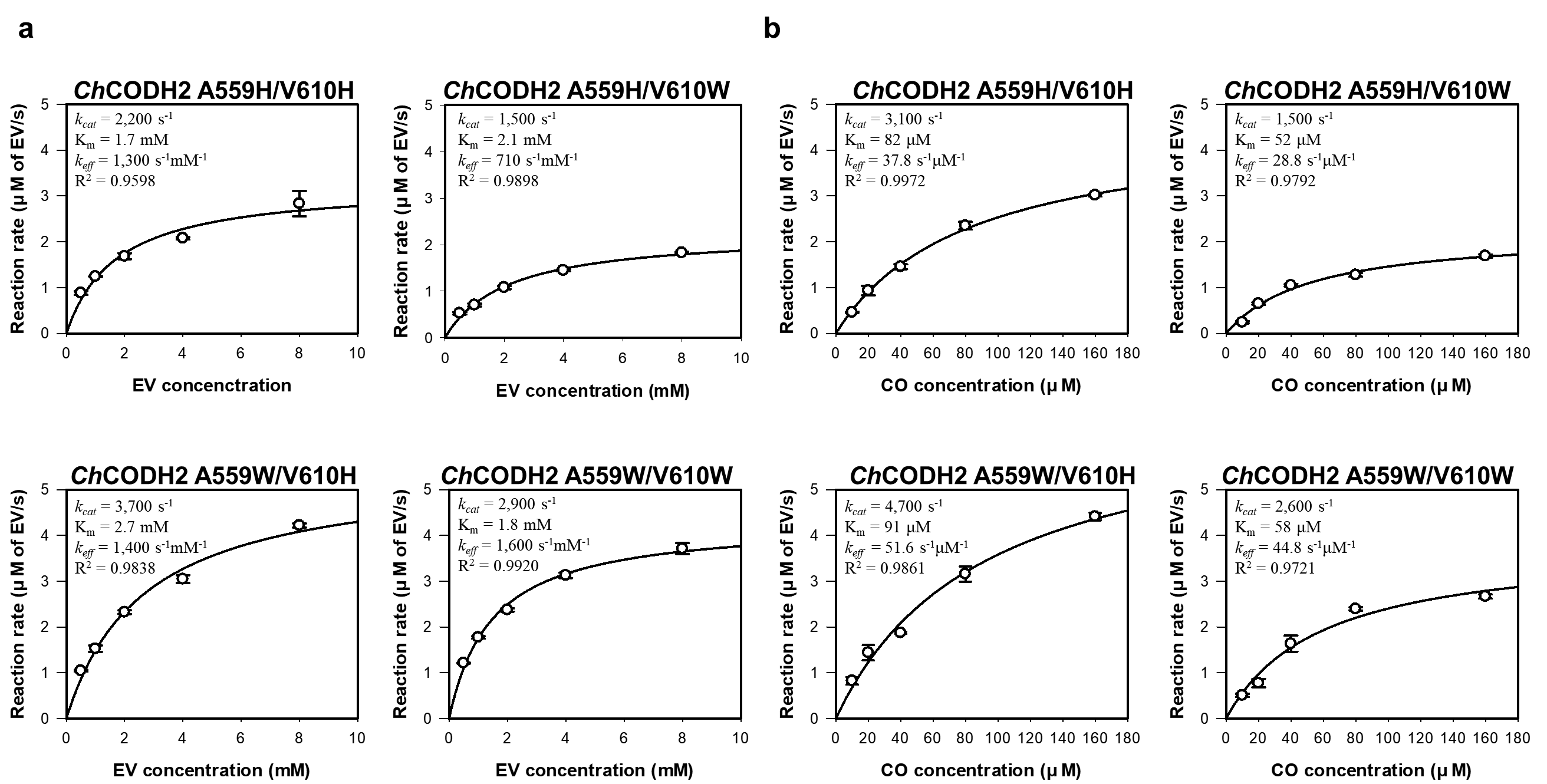
**

**Figure S14 | Nonlinear hyperbolic kinetic profiles across *Ch*CODH2 variants.** Catalytic properties of *Ch*CODH2 mutants for EV and CO were estimated from the non-linear regression method. (a) *Ch*CODH2 mutants for EV. (b) *Ch*CODH2 mutants for CO. The values of *^app^k*_cat_ were calculated from *V*_max_ for EV and CO. The data represent the mean ± S.D. determined from *n* = 3 independent experiments.

**
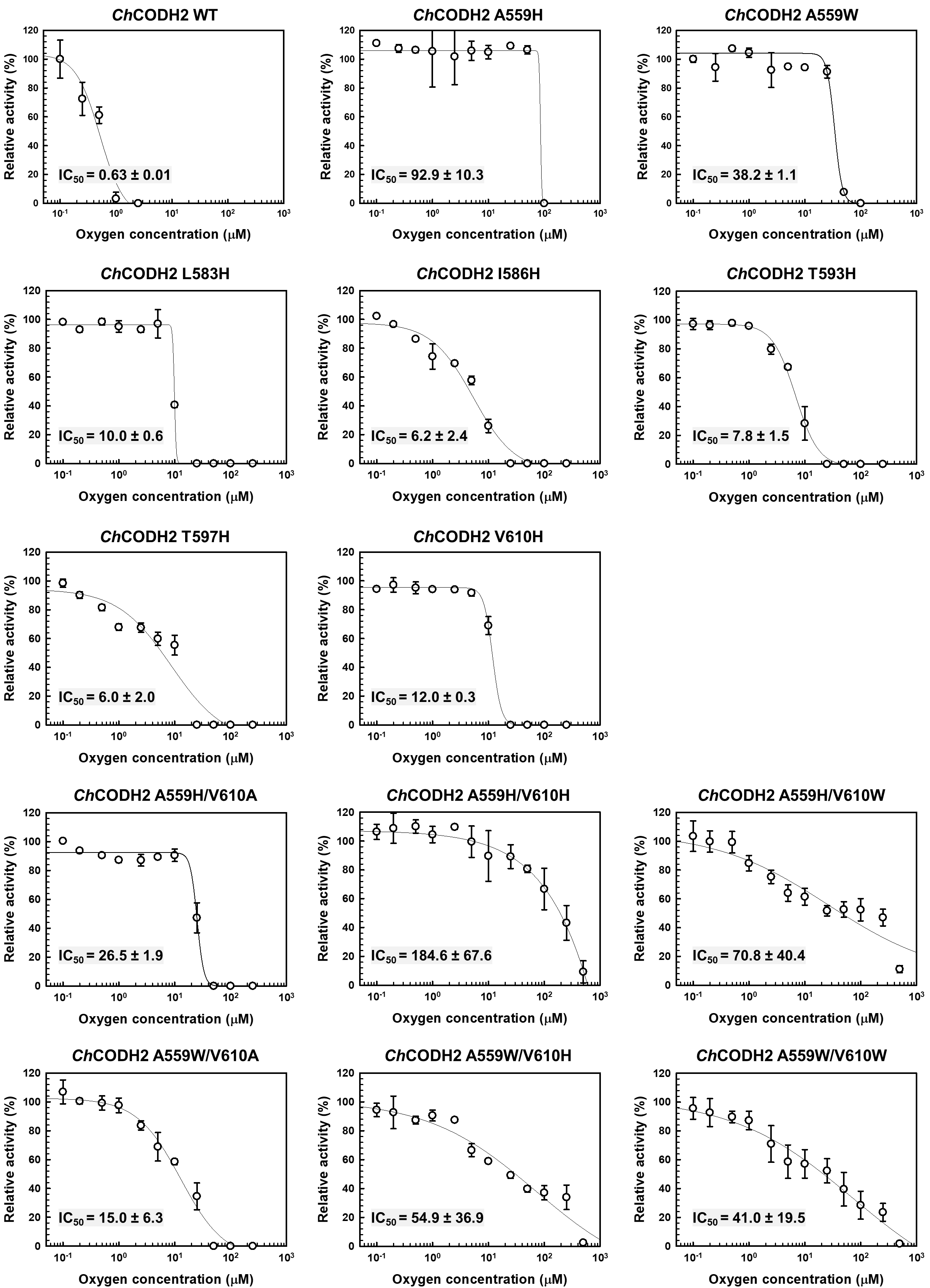
**

**Figure S15 | Determination of half-maximal inhibitory oxygen concentration (IC_50_) in *Ch*CODH2 and variants.** The half-maximal inhibitory oxygen concentrations (IC_50_) values were calculated using nonlinear regressions (four-parameter logistic curve). The data represent the mean ± S.D., as determined from *n*= 3 independent experiments.
